# Single-cell Profiling Uncovers a *Muc4*-Expressing Metaplastic Gastric Cell Type Sustained by *Helicobacter pylori*-driven Inflammation

**DOI:** 10.1158/2767-9764.CRC-23-0142

**Published:** 2023-09-05

**Authors:** Valerie P. O'Brien, Yuqi Kang, Meera K. Shenoy, Greg Finak, William C. Young, Julien Dubrulle, Lisa Koch, Armando E. Rodriguez Martinez, Jeffery Williams, Elizabeth Donato, Surinder K. Batra, Cecilia C.S. Yeung, William M. Grady, Meghan A. Koch, Raphael Gottardo, Nina R. Salama

**Affiliations:** 1 Human Biology Division, Fred Hutchinson Cancer Center, Seattle, Washington.; 2 Basic Sciences Division, Fred Hutchinson Cancer Center, Seattle, Washington.; 3 Vaccine and Infectious Disease Division, Fred Hutchinson Cancer Center, Seattle, Washington.; 4 Shared Resources, Fred Hutchinson Cancer Center, Seattle, Washington.; 5 Division of Gastrointestinal and Hepatic Pathology, University of Washington Medical Center, Seattle, Washington.; 6 Translational Science and Therapeutics Division, Fred Hutchinson Cancer Center, Seattle, Washington.; 7 Department of Biochemistry and Molecular Biology, University of Nebraska Medical Center, Omaha, Nebraska.; 8 Department of Laboratory Medicine and Pathology, University of Washington Medical Center, Seattle, Washington.; 9 Department of Medicine, University of Washington School of Medicine, Seattle, Washington.; 10 Department of Immunology, University of Washington, Seattle, Washington.; 11 Department of Microbiology, University of Washington, Seattle, Washington.

## Abstract

Mechanisms for *Helicobacter pylori* (*Hp*)-driven stomach cancer are not fully understood. In a transgenic mouse model of gastric preneoplasia, concomitant *Hp* infection and induction of constitutively active KRAS (*Hp*+KRAS+) alters metaplasia phenotypes and elicits greater inflammation than either perturbation alone. Gastric single-cell RNA sequencing showed that *Hp*+KRAS+ mice had a large population of metaplastic pit cells that expressed the intestinal mucin *Muc4* and the growth factor amphiregulin*.* Flow cytometry and IHC-based immune profiling revealed that metaplastic pit cells were associated with macrophage and T-cell inflammation. Accordingly, expansion of metaplastic pit cells was prevented by gastric immunosuppression and reversed by antibiotic eradication of *Hp*. Finally, MUC4 expression was significantly associated with proliferation in human gastric cancer samples. These studies identify an *Hp*-associated metaplastic pit cell lineage, also found in human gastric cancer tissues, whose expansion is driven by *Hp*-dependent inflammation.

**Significance:**

Using a mouse model, we have delineated metaplastic pit cells as a precancerous cell type whose expansion requires *Hp*-driven inflammation. In humans, metaplastic pit cells show enhanced proliferation as well as enrichment in precancer and early cancer tissues, highlighting an early step in the gastric metaplasia to cancer cascade.

## Introduction

Gastric adenocarcinoma is a canonical example of chronic inflammation–associated carcinoma. About 80% of cases are attributable to infection with *Helicobacter pylori* (*Hp*; ref. 1), a bacterium that colonizes the stomach of half the world's population (2). *Hp* infection causes gastric inflammation (superficial gastritis). In some individuals, inflammation triggers the loss of gastric acid–producing parietal cells (chronic atrophic gastritis) and subsequent elevation of stomach pH. In response to parietal cell loss, digestive enzyme-producing chief cells (hypothesized to be a cell of origin of gastric intestinal metaplasia [IM; refs. 3, 4]) can transdifferentiate into metaplastic cells, which can precede the appearance of dysplasia and gastric cancer (5–11). Thus, *Hp* infection elicits the chronic inflammation that promotes gastric adenocarcinoma development, and eradication of *Hp* with antibiotics significantly reduces (but does not eliminate) the risk of gastric cancer development and recurrence (12–14). However, the exact mechanism(s) through which *Hp* infection and associated inflammation drive gastric disease remain poorly understood, which limits our ability to develop effective therapies to target this immunopathology.

In mice, key aspects of human preneoplastic progression, including the development of metaplasia with intestinal features (gastric intestinal metaplasia) and mild dysplasia, can be modeled by the induction of a constitutively active *Kras* allele in gastric chief cells (3). We previously found that concomitant *Hp* infection of these KRAS transgenic mice (*Hp*+KRAS+) elicited greater dysplasia compared with *Hp*−KRAS+ mice (15). This phenotype could be due to either *Hp* infection accelerating the disease trajectory that results from constitutively active KRAS, or *Hp* infection driving a different disease trajectory. To distinguish between these possibilities, we used single-cell RNA sequencing (scRNA-seq) to examine changes in cell populations that occur in the context of gastric intestinal metaplasia with and without *Hp* infection. We found that mice with concomitant chronic *Hp* infection and transgene-driven gastric intestinal metaplasia developed an abundant population of *Muc4*-expressing metaplastic pit cells that exhibited a genetic signature of inflammation and epithelial changes including epithelial-to-mesenchymal (EMT) transition, compared with mice without *Hp* infection. Sustained *Hp* infection and associated gastric inflammation were required for pit cell metaplasia. Studies of human samples revealed that metaplastic pit cells increase in abundance as gastric disease develops, and MUC4 expression is significantly associated with cell proliferation. Taken together, these studies define a novel cell state that is driven by *Hp*-mediated inflammation and associated with cell proliferation and gastric cancer.

## Materials and Methods

All mouse experiments were approved by the Fred Hutchinson Cancer Center Institutional Animal Care and Use Committee (protocol number 1531) and were performed in accordance with the recommendations in the NIH Guide for the Care and Use of Laboratory Animals. *Mist1-CreERT2 Tg/+,LSL-Kras*^G12D^*Tg/+* (“*Mist1-Kras*”) mice were described previously (3, 15). *Hp* strain PMSS1 or an isogenic Δ*cagE* mutant was cultured and mice were inoculated with 10^8^ colony-forming units (CFU) as described previously (15). For scRNA-seq, single-cell suspensions were generated using digestion at 4°C with protease from *Bacillus licheniformis* (Sigma P5380) using an established protocol (16), and cells were used for gel beads-in-emulsion (GEM) generation and barcoding using the Chromium Next GEM Single Cell 3ʹ Reagent Kits v3.1 and Nextera library preparation (both from 10x Genomics) according to the manufacturer's instructions. For flow cytometry analysis, lamina propria cells were isolated using Liberase TL (Roche) and stained cells were run on a BD FACSymphony A5 High-Parameter Cell Analyzer. Immunofluorescence microscopy to detect MUC4/Ki-67 and multiplex immune cell IHC studies to detect immune cells were performed as described previously (15). ISH was performed on fixed tissue sections using the RNAscope system (ACD-biotechne) in accordance with the manufacturer's instructions, using a Leica Bond RX autostainer. Human gastric tissues for constructing a tissue microarray (TMA) were obtained from the University of Washington Northwest BioSpecimen tissue repository, Seattle, WA, or from the Legacy Research Institute Tumor Bank, Portland, OR (the latter accessed through the Fred Hutch Specimen Acquisition Network). All procedures were reviewed by the Fred Hutchinson Cancer Center Institutional Review Board (IR8657) and were conducted in accordance with recognized ethical guidelines of the Declaration of Helsinki, Belmont Report and U.S. Common Rule. Three published scRNA-seq studies were used as a validation cohort (17–19). Additional information about the materials and methods in this study may be found in the Supplementary Materials and Methods section of the supporting information, and a list of antibodies and probes is given in Supplementary Table S8.

### Data Availability Statement

scRNA-seq data were deposited in Gene Expression Omnibus under the project accession number GSE224840. The accession numbers for the individual samples are: GSM7034070, GSM7034071, GSM7034072, GSM7034073, GSM7034074, GSM7034075, GSM7034076, and GSM7034077. Other data generated in this study are found in the Supplementary Tables or are available from the corresponding author upon request.

## Results

### scRNA-seq Revealed Heterogeneous Pit Cell Populations Associated with *Hp* Infection and Metaplasia Induction

**FIGURE 1:**
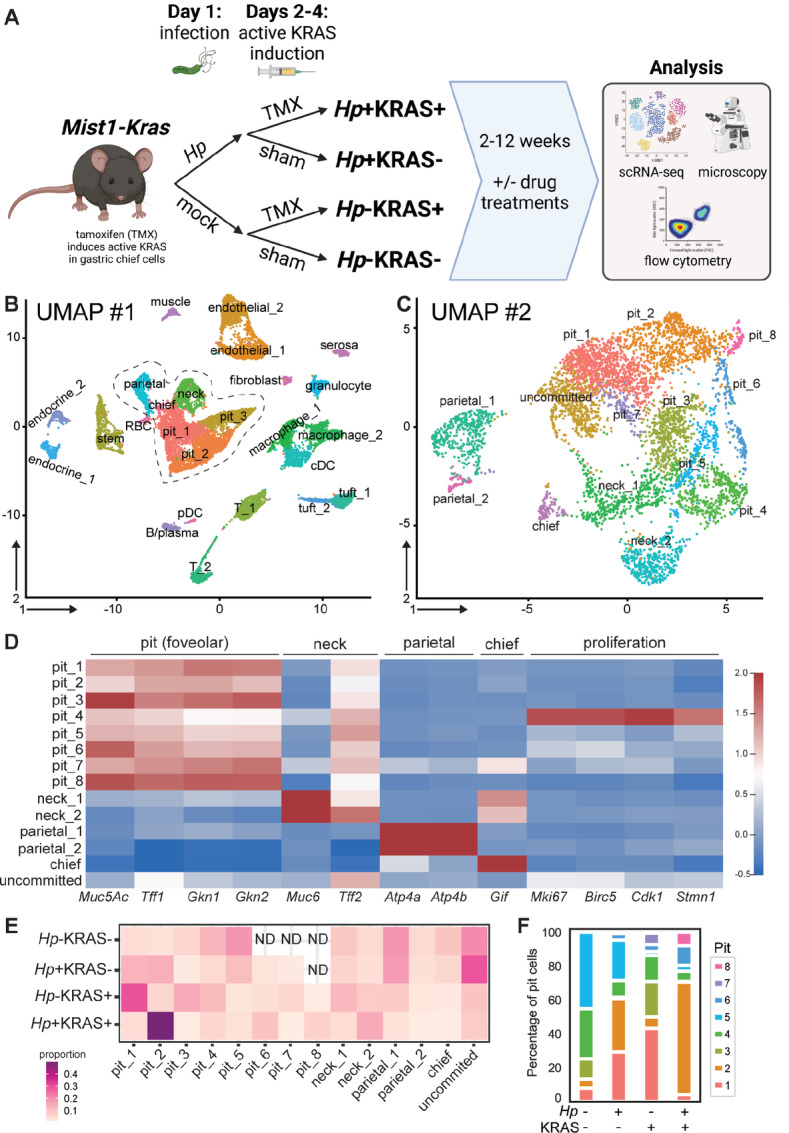
Gastric scRNA-seq reveals expansion of pit cell populations in *Hp*+KRAS+ mice. **A,** Overview and experimental timeline for mouse experiments, created with BioRender.com. TMX, tamoxifen. **B–F,** Mice ± *Hp* infection and ± constitutively active KRAS induction were used for gastric scRNA-seq. Corpus single-cell suspensions were prepared from: one *Hp*−KRAS+ and one *Hp*+KRAS+ mouse at 6 weeks; and at 12 weeks, three *Hp*−KRAS− and two *Hp*+KRAS− (each pooled into one sample), one individual (never cryopreserved) and two pooled *Hp*−KRAS+ mice, and one individual (never cryopreserved) and two pooled *Hp*+KRAS+ mice. **B,** Cluster analyses followed by UMAP visualization of the entire dataset yielded 25 clusters, including epithelial and immune cell types, which were manually annotated on the basis of gene expression. Endocrine, enteroendocrine cell; cDC, conventional dendritic cell; RBC, erythrocyte/reticulocyte; pDC, plasmacytoid dendritic cell. **C,** Cells from the 12 week timepoint in the central epithelial cell megacluster, outlined with the dotted line in B, were subjected to reclustering followed by UMAP visualization, yielding 14 clusters that were manually annotated on the basis of gene expression. **D,** The normalized expression of marker genes for the indicated gastric epithelial cell types is shown for the 14 epithelial clusters from UMAP #2 in C. **E,** The estimated proportion of each of the 14 epithelial clusters detected in the pilot scRNA-seq experiment in the indicated treatment groups at 12 weeks is shown (rows add to 100%). ND, cell cluster was not detected in the indicated treatment group. **F,** The distribution of pit cell clusters within each treatment group in E is shown.

We previously found that in *Mist1-CreERT2, LSL-Kras*^G12D^ (“*Mist1-Kras”*) mice, concomitant *Hp* infection and metaplasia driven by constitutively active KRAS induction led to tissue changes including altered gastric gland architecture, changes to metaplasia marker expression, increased dysplasia and severe inflammation within 12 weeks (15). To obtain an unbiased view of gene expression changes in gastric cell types, we conducted an exploratory scRNA-seq experiment with mice ± *Hp*, ± KRAS at 12 weeks (Fig. 1A; Supplementary Table S1). We also analyzed tissues from KRAS+ mice ± *Hp* at 6 weeks to probe how cell populations changed over time. Because constitutively active KRAS is targeted to the corpus (body) of the stomach in this model, the gastric forestomach and antrum were discarded prior to sample processing. This experiment yielded 22,050 cells, >75% of which were viable based on mitochondrial gene filtering. Cluster analyses followed by visualization with Uniform Manifold Approximation and Projection (UMAP) identified 25 cell clusters, which we manually annotated on the basis of marker gene expression (UMAP #1, Fig. 1B; Supplementary Figs. S1 and S2; Supplementary Table S2). The gene expression profiles from the two samples collected at 6 weeks (from 2 different mice) were largely similar to those from samples collected at 12 weeks (Supplementary Figs. S1 and S2). We detected several cell lineages, including putative immune cells (e.g., macrophages and T cells) as well as non-hematopoietic cells (e.g., endothelial and muscle cells). We focused on analyzing gastric epithelial cells to address our overarching question of whether *Hp*+KRAS+ mice had an altered or accelerated disease trajectory compared with *Hp*−KRAS+ mice. Glands in the gastric corpus are comprised of a variety of epithelial cell types, including acid-producing parietal cells, digestive enzyme-producing chief cells, and two mucus-producing cell types: pit (foveolar) cells at the surface and neck cells in the middle of the glands. Each of these cell types were found in a large epithelial “megacluster” (Fig. 1B, dashed lines). We observed three clusters within this megacluster that expressed the mucin and classical pit cell marker *Muc5Ac,* which we annotated as pit_1, pit_2, and pit_3.

To better discriminate among these gastric epithelial cell types and compare their abundance across all treatment groups, we reclustered the cells in the central epithelial megacluster from the 12 week timepoint alone. This analysis yielded 14 clusters that we manually annotated on the basis of gene expression (UMAP #2, Fig. 1C; Supplementary Table S3). Most of the clusters expressed low to moderate levels of the trefoil factor *Tff2*, which is expressed by several gastric corpus epithelial cell types (ref. 20; Fig. 1D). One cluster expressed *Tff2* but no other known marker genes, suggesting it may be an uncommitted or progenitor cell type. Further analysis showed that the expression of specific ribosomal genes differentiated this cluster (Supplementary Fig. S3; Supplementary Table S4). Eight of the clusters expressed classical pit cell genes (*Muc5Ac*, trefoil factor *Tff1* and the gastrokines *Gkn1* and *Gkn2*) and we annotated them as pit_1 through pit_8 (Fig. 1D). The pit_4 cluster also expressed proliferation markers (*Mki67*, *Birc5*, *Cdk1,* and *Stmn1*), suggesting these cells may be pit cell progenitors.

The abundance of the different epithelial clusters varied in the different treatment groups, which could reflect biological changes among the treatment groups and/or could be due to variability inherent in sample processing (Fig. 1E; Supplementary Fig. S4). Parietal and chief cells were less abundant in *Hp*−KRAS+ mice and least abundant in *Hp*+KRAS+ mice, consistent with our previous analysis of tissues from these mice (15). To explore pit cell heterogeneity, we determined the proportion of all pit cells annotated as each individual cluster (Fig. 1F). The predominant pit cell clusters in each treatment group were: pit_5 (45%) and pit_4 (30%) in *Hp*−KRAS− (healthy) mice; pit_2 (32%) and pit_1 (29%) in *Hp*+KRAS− mice; pit_1 (43%) in *Hp*−KRAS+ mice; and pit_2 (66%) in *Hp*+KRAS+ mice. Thus, pit cells may be a heterogeneous population whose composition can dynamically change due to infection and/or metaplasia.

### *Hp*+KRAS+ Mice have Abundant *Muc4*-expressing Pit Cells

**FIGURE 2:**
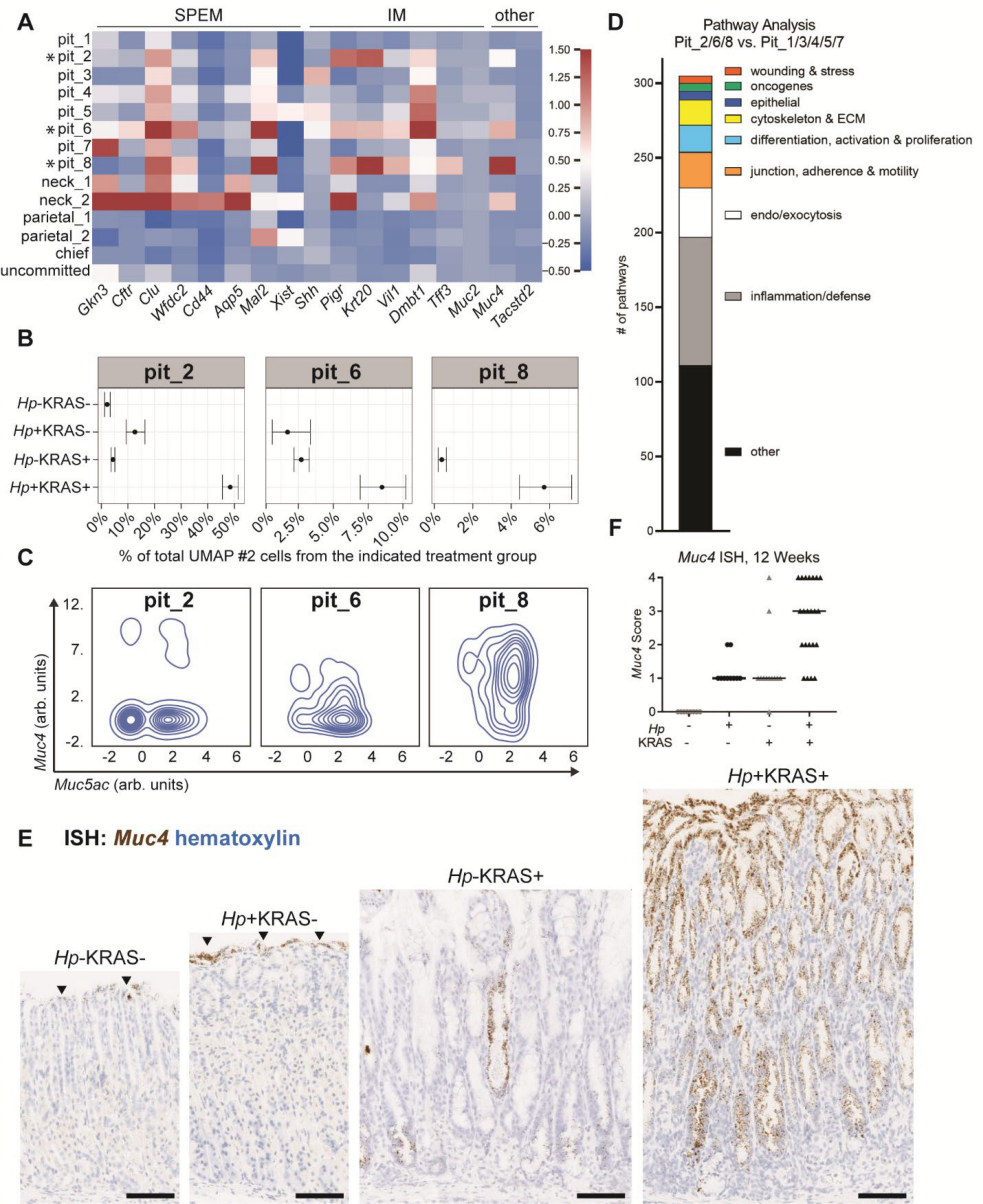
*Hp*+KRAS+ mice have abundant metaplastic pit cells that express the intestinal mucin *Muc4*. **A,** The normalized expression of the indicated metaplasia and dysplasia genes is given for the cells from UMAP #2 (Fig. 1C). Asterisks indicate metaplastic pit cells. **B,** The estimated proportion of cells from the scRNA-seq experiment that were assigned to the indicated pit cell clusters is shown. Each datapoint shows the total number of estimated cells of that subcluster for the given treatment group, reported as the percentage of all cells from that treatment group in UMAP #2. Error bars represent the confidence interval that a given percentage of cells would be identified as the given cluster type based on their observed distribution. **C,** The contour plots show *Muc5Ac* (*x*-axis) and *Muc4* (*y*-axis) expression in the indicated pit cell clusters from UMAP #2. Expression is given as arbitrary units and negative values are due to data scaling for visualization purposes. **D,** Gene set enrichment analysis was performed to assess biological pathways and processes enriched in pit_2, pit_6, and pit_8 cells relative to the five other pit cell clusters, using Hallmark, KEGG, and GO pathway datasets from the MSigDB database. **E** and **F,** ISH was used to detect the intestinal mucin *Muc4* (brown) in gastric corpus tissue (counterstained with hematoxylin, blue) from the indicated treatment groups at 12 weeks. **E,** Representative images are shown. Scale bars, 100 μm. Arrows show *Muc4* expression at the epithelial surface in KRAS− samples. **F,** Tissues were scored in a blinded fashion. *N* = 5 independent experiments were performed with *n* = 3–8 mice per group. Data represent actual values from each individual mouse and bars indicate the median values.

Because our data suggested that pit cell populations can differ between mice with and without metaplasia, we queried whether any pit cell clusters or other epithelial cell types expressed metaplasia-associated genes (Fig. 2A). The neck_2 cluster expressed several genes associated with a type of gastric adenocarcinoma–associated metaplasia called spasmolytic polypeptide-expressing metaplasia (SPEM; refs. 21–23), including gastrokine *Gkn3*, the chloride channel *Cftr*, clusterin (*Clu*), *Wfcd2* (HE4), the cell surface glycoprotein *Cd44*, and the water channel protein *Aqp5*, but had low expression of the SPEM markers *Mal2* (a proteolipid family member) and the non-coding RNA *Xist*. This neck_2 cluster was most abundant in *Hp*+KRAS+ mice (Fig. 1E; Supplementary Fig. S4). This observation is in concordance with our previous finding that *Hp*+KRAS+ mice had increased SPEM compared with *Hp*−KRAS+ mice at 12 weeks (15). We next investigated the expression of IM genes (24–27). Sonic hedgehog (*Shh*) was expressed at low levels in pit_3, pit_5, and pit_6 cells. Along with the neck_2 cluster, pit_2, pit_6, and pit_8 stood out from the other clusters due to low or moderate expression of several IM-related genes: the polymeric immunoglobulin receptor *Pigr*, keratin *Krt20*, and villin *Vil1*. The IM-associated, candidate tumor suppressor glycoprotein *Dmbt1* was expressed by these clusters and several additional pit cell clusters, and the IM marker trefoil factor *Tff3* was expressed in pit_8 cells. The IM-associated mucin *Muc2* was not expressed in any cell type, but another intestinal mucin, *Muc4*, was highly expressed in the pit_8 cluster and moderately in pit_2, pit_6, and neck_2. Finally, *Tacstd2* (TROP2), a dysplasia and stem cell marker (28), was not detected in any of the UMAP #2 cells, though it was detected in the stem cell cluster in UMAP #1 (Supplementary Fig. S5).

We previously observed that *Hp*+KRAS+ mice had increased pit cell hyperplasia compared with *Hp*−KRAS+ mice (15). However, this analysis was based on tissue scoring, so we were unable to determine whether the expanded pit cell populations were genetically similar to or different from classical pit cells. Here we observed that the pit_2, pit_6, and pit_8 clusters were all expanded in *Hp*+KRAS+ mice (Figs. 1F and 2B); pit_6 and pit_8 were not detected in healthy mice. We detected *Muc4* expression in 8% of cells in pit_2, 36% in pit_6, and 84% in pit_8 (Fig. 2C). To probe what biological processes and pathways differentiate these clusters from the other pit-related clusters, we performed gene set enrichment analysis with the Gene Ontology (GO), Kyoto Encyclopedia of Genes and Genomes (KEGG), and Hallmark databases, comparing all cells in the pit_2, pit_6, and pit_8 subclusters with all cells in the other five pit cell clusters for the entire dataset (all mice; Fig. 2D; Supplementary Table S5). A total of 305 pathways and processes were enriched in pit_2/6/8 cells, which we categorized on the basis of function (Fig. 2D). We previously found that *Hp*+KRAS+ mice had severe inflammation at 12 weeks (15), and accordingly, the most common categorized pathways (*n* = 85) pertained to inflammation and host defense, including interferon response, TNFα signaling via NFκB, complement, response to cytokines and IL2/STAT5 signaling. Another 24 pertained to cell-cell junctions, adherence and motility and 17 pertained to the cytoskeleton and extracellular matrix, suggesting that pit_2/6/8 cells may be undergoing cellular remodeling. Eighteen pathways concerned cell differentiation, activation, or proliferation, which could indicate that pit_2/6/8 cells were undergoing cell fate changes. Other enriched pathways of interest were associated with epithelial cells (six pathways including epithelial-mesenchymal transition [EMT], a hallmark of cancer development) and wound healing and stress (five pathways), which could suggest that these cells are generated by, or in response to, epithelial damage. Finally, five pathways involving gastric cancer–associated oncogenes were upregulated: KRAS signaling, the P53 pathway, and ErbB signaling. Because pit_2, pit_6, and pit_8 cells were most abundant in *Hp*+KRAS+ mice and expressed metaplasia-associated genes and pathways related to cellular remodeling and fate changes, EMT, and oncogenesis, we termed these populations metaplastic pit cells.

To explore metaplastic pit cell abundance and localization, we focused on *Muc4*, which encodes an intestinal goblet cell mucin that in mice is normally expressed in cells of the first gland adjacent to the forestomach-glandular stomach junction, and sparsely at the luminal surface of the glandular stomach (29, 30). *In situ* hybridization (ISH) showed robust *Muc4* expression throughout the corpus of *Hp*+KRAS+ mice at 12 weeks (Fig. 2E), which we confirmed using spatial transcriptomics (Supplementary Fig. S6). *Muc4*-expressing pit cells were found in *Hp*+KRAS+ mice by 6 weeks using scRNA-seq and ISH (Supplementary Fig. S7A and S7B). We thus used *Muc4* expression as a proxy for metaplastic pit cells. In a blinded semiquantitative scoring of mice at 12 weeks (see Supplementary Materials and Methods), *Muc4* expression was greater in *Hp*+KRAS+ mice than in any other group (Fig. 2F). To test whether *Muc4* expression was driven by the *Hp cag* type IV secretion system (T4SS), which injects inflammatory and oncogenic bacterial factors into gastric epithelial cells (31, 32) and which is associated with increased risk of gastric disease (8), we infected mice with an isogenic mutant that cannot assemble the T4SS, *Hp*Δ*cagE*. In these mice, *Muc4* expression had an intermediate phenotype (Supplementary Fig. S7B): lower than in mice infected with wild-type *Hp* but still higher than in mock-infected mice, suggesting both *cag* T4SS-dependent and independent contributions to *Muc4* expression. Finally, *Muc4* expression did not correlate with gastric *Hp* bacterial burden (Supplementary Fig. S7C). In summary, the expansion of *Muc4*-expressing metaplastic pit cells requires the combination of *Hp* infection and active KRAS; neither *Hp* infection nor metaplasia induction alone is sufficient to drive this phenotype within the 12 week experimental time frame.

### Metaplastic Pit Cell Development Requires Sustained Gastric Inflammation

**FIGURE 3:**
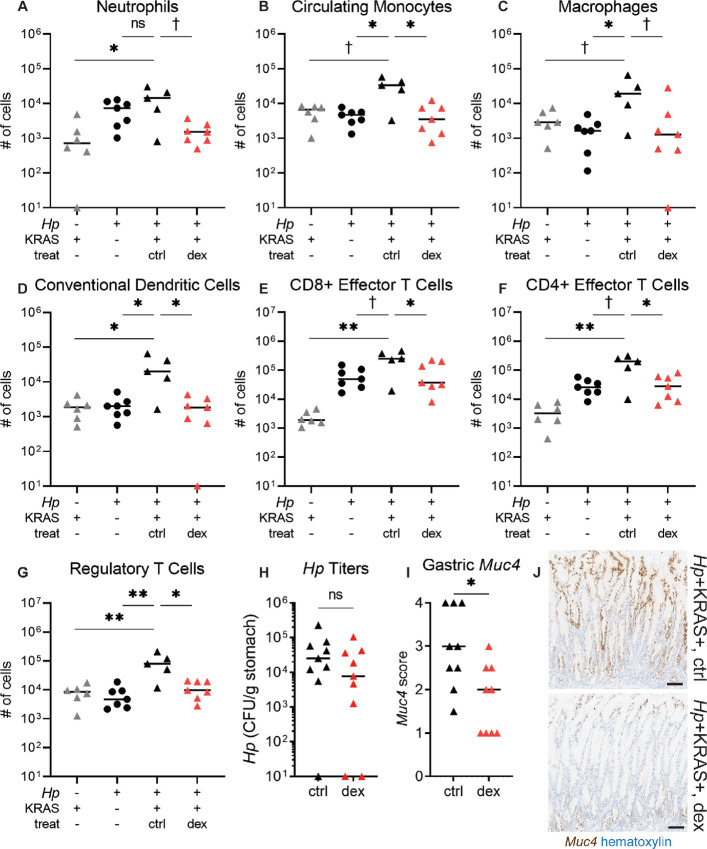
Gastric immunosuppression prevents metaplastic pit cell expansion. **A–G,** Flow cytometry was performed at 6 weeks using *Hp*−KRAS+ and *Hp*+KRAS− mice, and *Hp*+KRAS+ mice who received either oral dexamethasone (“dex”) starting at 2 weeks or water alone (control, “ctrl”). Graphs indicate the number of live leukocytes isolated from the gastric lamina propria of the indicated mouse groups. CD4−CD8−Ly6C+Ly6G+ neutrophils (A), CD11b variable Ly6C+ MHC II– circulating monocytes (B), CD64+F4/80+ macrophages (C), CD11c high MHC II high conventional dendritic cells (D), CD3+CD8α+CD103+CD44+ effector T cells (E), CD3+CD4+CD103+CD44+ effector T cells (**F**), CD3+CD4+CD103+CD44+FoxP3+ regulatory T cells (**G**). *N* = 2 independent experiments were conducted and data from one experiment is shown. Datapoints represent actual values for each individual mouse, zeroes are plotted at the limit of detection (10 cells), and bars indicate median values. **H–J,***Hp*+KRAS+ mice that were control-treated or dexamethasone-treated are shown. **H,***Hp* was cultured from stomach homogenate supernatants at time of euthanasia. CFU, colony-forming units. Zeroes are plotted at the limit of detection (10 CFU). **I** and **J,***Muc4* (brown) was detected by ISH in *N* = 2 independent experiments with *n* = 3–6 mice per group. I, *Muc4* expression was scored in a blinded fashion. J, Representative images are shown. Scale bars, 100 μm. †, *P* < 0.10; *, *P* ≤ 0.05; **, *P* < 0.01; ns, not significant, Mann–Whitney *U* test.

Because metaplastic pit cells were enriched for transcripts associated with immune-related pathways, we explored whether inflammation was required for their expansion. It was previously shown that group 2 innate lymphoid cells could promote gastric intestinal metaplasia as a wound-healing response after tissue injury (33). However, the type 2 cytokines *Il4*, *Il5*, *Il9,* and *Il13* were not detected in our scRNA-seq dataset, and type 2 inflammatory pathways were not uncovered in our gene set enrichment analysis (Supplementary Table S5). As well, IL4 levels in gastric homogenate supernatants did not differ among the treatment groups, whereas the classical type 1 cytokine IFNγ was elevated in mice with metaplastic pit cells (Supplementary Fig. S8). Thus, we performed flow cytometry (Supplementary Figs. S9 and S10) to profile immune cell populations in the gastric lamina propria at 6 weeks, a timepoint at which metaplasia and proliferation phenotypes in *Hp*+KRAS+ mice have already begun to diverge from *Hp*−KRAS+ mice (Supplementary Fig. S7; ref. 15). We compared *Hp*+KRAS+ mice with *Hp*+KRAS− and *Hp*−KRAS+ mice (Fig. 3A–H), reasoning that the immune cells involved in metaplastic pit cell accumulation should only be increased in *Hp*+KRAS+ mice. Neutrophils were elevated in both *Hp*+KRAS+ and *Hp*+KRAS− mice compared with *Hp*−KRAS+ mice (Fig. 3A), indicating that their accumulation was due to *Hp* infection alone. However, circulating monocytes, macrophages, and conventional dendritic cells were all significantly increased in *Hp*+KRAS+ mice compared with both control groups (Fig. 3B–D). Among lymphocytes, we saw increased CD8^+^ cytotoxic T cells and CD4^+^ helper T cells (Th) cells in *Hp*+KRAS+ mice. The majority of these cells were CD103^+^, consistent with a tissue-resident phenotype, and had high expression of CD44, consistent with an effector phenotype (Fig. 3E and F). As well, *Hp*+KRAS+ mice had the greatest numbers of regulatory T cells (Fig. 3G). Thus, *Hp*+KRAS+ mice have significant increases in gastric lamina propria myeloid cells and effector T cells, but not neutrophils.

To test whether inflammation was necessary for metaplastic pit cell expansion, we treated *Hp*+KRAS+ mice with the immunosuppressive glucocorticoid dexamethasone. Mice received dexamethasone *ad libitum* in the drinking water starting at 2 weeks and were euthanized at 6 weeks. *Hp* bacterial loads were not significantly different between the two groups (Fig. 3H). As expected, dexamethasone treatment significantly reduced the accumulation of gastric immune cell populations compared with control-treated *Hp*+KRAS+ mice (Fig. 3A–G). Importantly, gastric *Muc4* expression was also significantly reduced, though not eliminated, in dexamethasone-treated mice (Fig. 3I and J). Thus, gastric inflammation is necessary for metaplastic pit cell development in *Hp*+KRAS+ mice and is primarily comprised of increased gastric macrophages and T cells.

**FIGURE 4:**
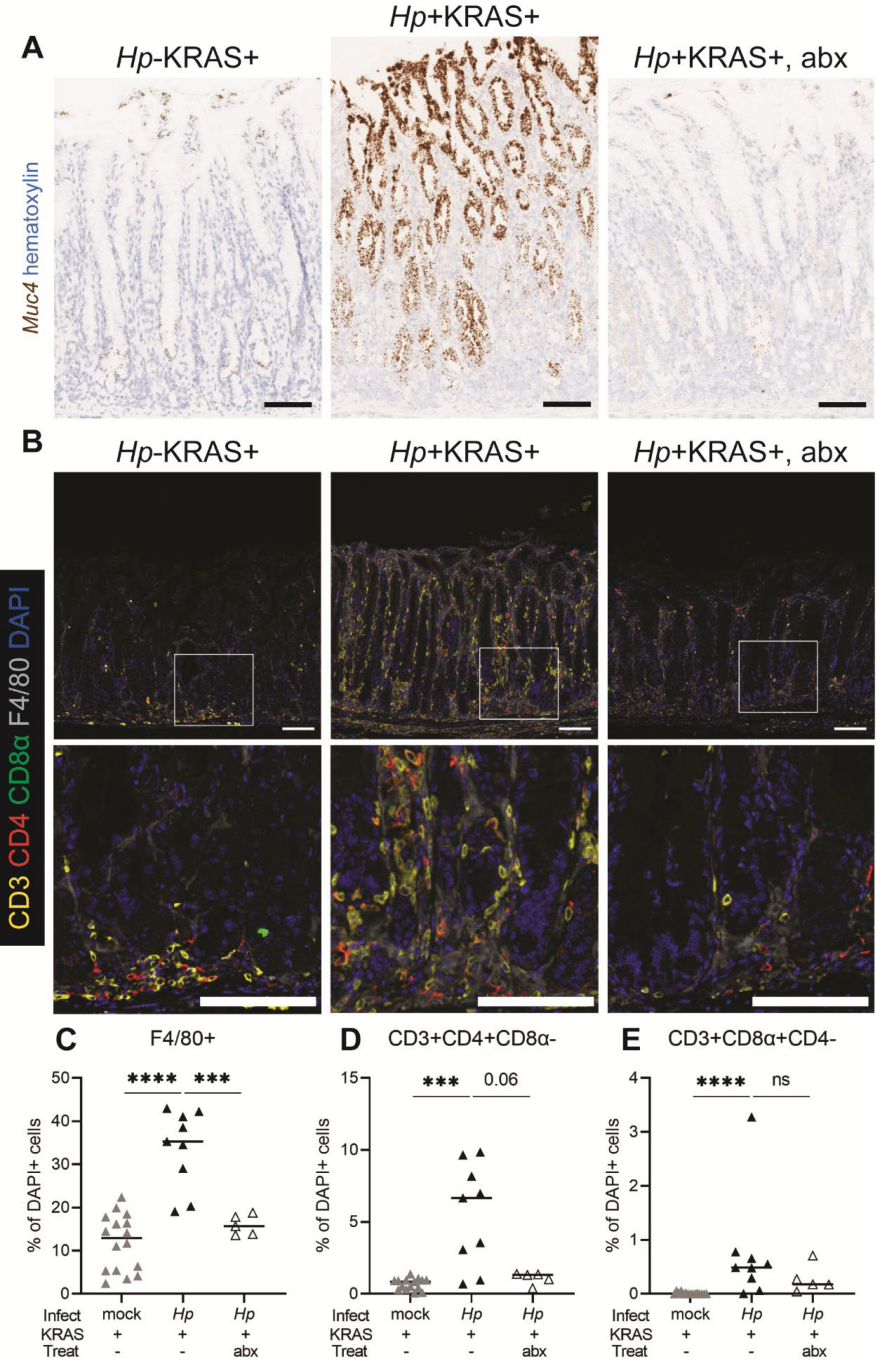
Antibiotic eradication of *Hp* reverses metaplastic pit cell expansion. *Muc4* and gastric inflammation were assessed 12 weeks after KRAS induction in the following groups: *Hp*−KRAS+ mice; *Hp*+KRAS+ mice; and *Hp*+KRAS+ mice treated with antibiotics (“abx”) from weeks 6 to 8. **A,** ISH was used to detect *Muc4* (brown) in the corpus at 12 weeks and representative images are shown. Scale bars, 100 μm. **B–E,** IHC was used to detect the indicated immune cell populations in gastric corpus tissue at 12 weeks. Data are from *N* = 2 independent experiments with *n* = 5–10 mice per group. **B,** Representative images of the gastric corpus are shown. CD3 (yellow) indicates T cells, CD4 (red) indicates Th cells, CD8α (green) indicates cytotoxic T cells, F4/80 (gray) indicates macrophages and DAPI (blue) indicates nuclei. Higher magnification images of the boxed regions are shown in the second row. Scale bars, 100 μm. **C–E,** The indicated immune cell populations were detected by IHC and quantified in each mouse using HALO software in *N* = 2 experiments. Each datapoint is the average quantitation in two fields of view for an individual mouse and bars represent the medians. ***, *P* < 0.001; ****, *P* < 0.0001; Mann–Whitney *U* test; ns, not significant.

To confirm that *Hp* was responsible for inducing inflammation that drove metaplastic pit cell expansion, we tested samples from *Hp*+KRAS+ mice given antibiotics from 6 to 8 weeks after infection and constitutively active KRAS induction (15). *Muc4* expression was strongly reduced at 12 weeks in antibiotic-treated mice (Fig. 4A), again supporting our conclusion that metaplastic pit cell expansion requires sustained, *Hp*-driven inflammation.

One drawback to flow cytometry is the inability to visualize immune cell localization and distribution *in situ*. To visualize and further characterize gastric immunity in each mouse group, we used multiplex immunohistochemistry (IHC) (Fig. 4B; Supplementary Fig. S11). Because our flow cytometry data (Fig. 3) associated metaplastic pit cell expansion with an increased accumulation of macrophages and T cells in *Hp*+Kras+ mice, we focused on these immune cell populations. Each of these cell types was less abundant in *Hp*−KRAS+ mice compared with *Hp*+KRAS+ mice. Quantitative tissue analysis using HALO software revealed that antibiotic treatment of *Hp*+KRAS+ mice caused a reduction in macrophages (F4/80+, *P* < 0.001; Fig. 4C) and Th cells (CD3^+^ CD4^+^CD8α^−^, *P* = 0.06; Fig. 4D), but cytotoxic T cells (CD3^+^CD8α^+^CD4^−^) were not significantly impacted by antibiotic treatment (*P* = 0.30; Fig. 4E). We observed that macrophages were localized throughout the lamina propria in all mouse groups. However, T cells were located throughout the lamina propria only in *Hp*+KRAS+ mice, whereas they were largely restricted to the base of the gastric glands in the other treatment groups (Fig. 4B), suggesting that T cell inflammation may impact a broader range of epithelial cell types in *Hp*+KRAS+ mice. Thus, metaplastic pit cell expansion requires sustained, *Hp*-driven gastric inflammation.

### Metaplastic Pit Cells Express the EGFR Ligand Amphiregulin

**FIGURE 5:**
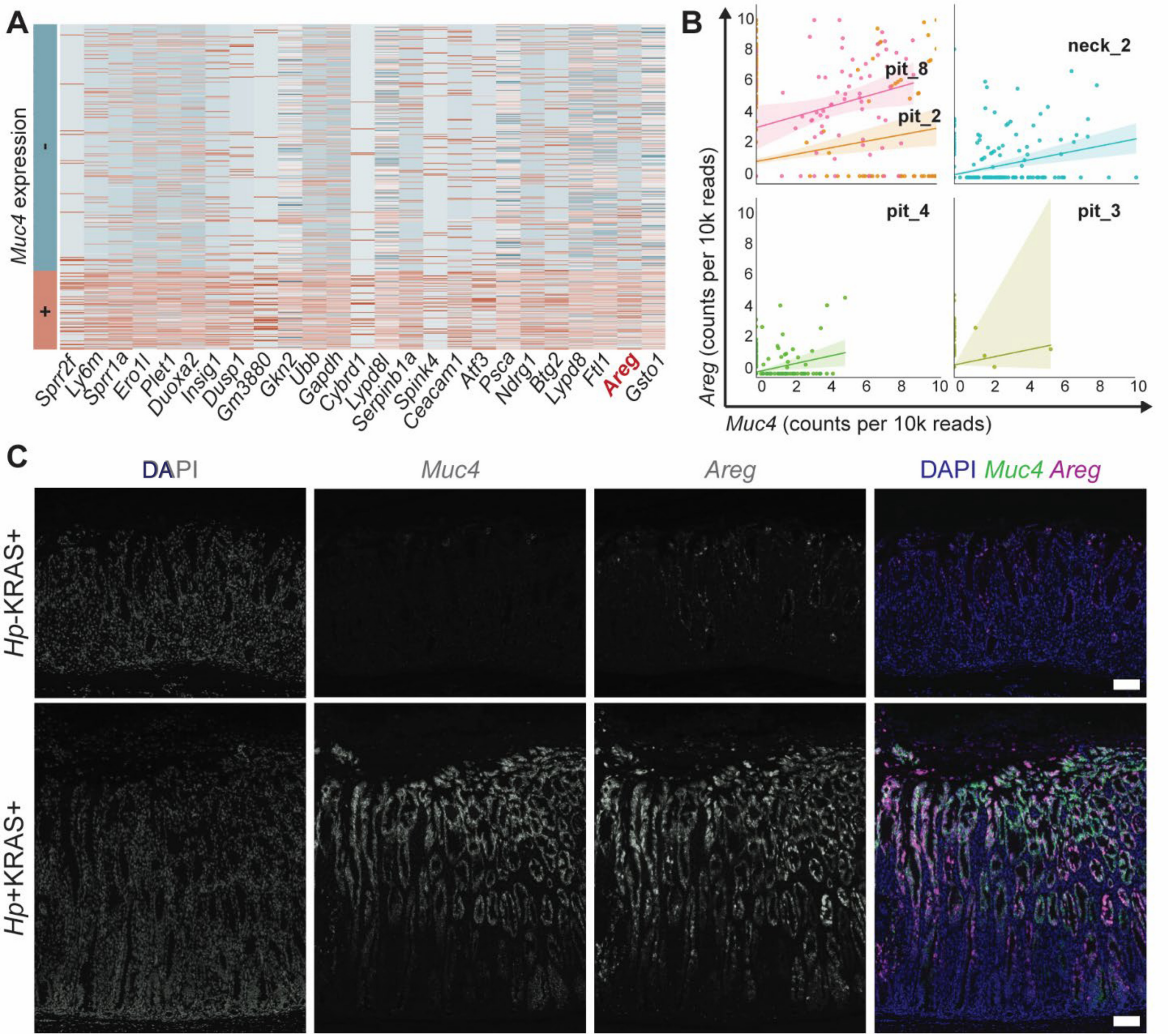
The EGFR ligand amphiregulin is another marker of metaplastic pit cells. **A,** The heat map shows the normalized expression of the top 25 genes that are most significantly differentially expressed between *Muc4*-expressing (bottom) and non-expressing (top) metaplastic pit cells (pit_2, pit_6, and pit_8 cells). Genes are ranked from left to right in order of statistical significance. **B,***Muc4* and *Areg* coexpression was assessed for each cluster in UMAP #2. Only the five indicated clusters had a Pearson correlation *P* value < 0.05. The expression of *Muc4* and *Areg* in counts per 10,000 reads is plotted for each cell in the given cluster. Trend lines are plotted and shading indicates the confidence interval of the linear estimation. **C,** Fluorescent ISH was used to detect *Muc4* (green) and *Areg* (magenta) from representative mice at 12 weeks; DAPI (blue) indicates nuclei and single channel images are shown in grayscale. Scale bars, 100 μm.

We next explored whether *Muc4* expression was associated with other genes that could contribute to the tissue changes observed in *Hp*+KRAS+ mice. We determined the genes most strongly correlated with *Muc4* expression in metaplastic pit cells (Fig. 5A) and found that one of the most strongly positively associated genes was *Areg* (amphiregulin), an EGFR ligand that promotes epithelial cell growth and wound healing. To validate *Areg* as a second marker of metaplastic pit cells, we first assessed *Muc4* and *Areg* coexpression in the epithelial compartment by assessing each cluster in UMAP #2. Five clusters had a Pearson correlation *P* value < 0.05 indicating statistically significant coexpression (Fig. 5B). Expression of each marker was highest in pit_8 cells followed by pit_2 cells. Among other epithelial cell types, some neck_2 cells (which are likely SPEM cells, Fig. 2A) as well as a few pit_3 and pit_4 cells also coexpressed these genes. Spatial transcriptomics revealed that the proportion of tissue spots that coexpressed *Muc4* and *Areg* in each sample was: 3.3% in *Hp*−KRAS−, 1.4% in *Hp*+KRAS-, 7.5% in *Hp*−KRAS+, and 17.8% in *Hp*+KRAS+ (Supplementary Fig. S6). Both *Muc4* and *Areg* were rarely expressed in non-epithelial cells (Supplementary Fig. S5). Finally, fluorescent ISH confirmed that *Muc4* and *Areg* strongly colocalized in *Hp*+KRAS+ mice at 12 weeks (Fig. 5C). Thus, metaplastic pit cells are marked not only by *Muc4* expression but also by *Areg* expression.

### MUC4 is Associated with Cell Proliferation in Gastric Cancer

**FIGURE 6:**
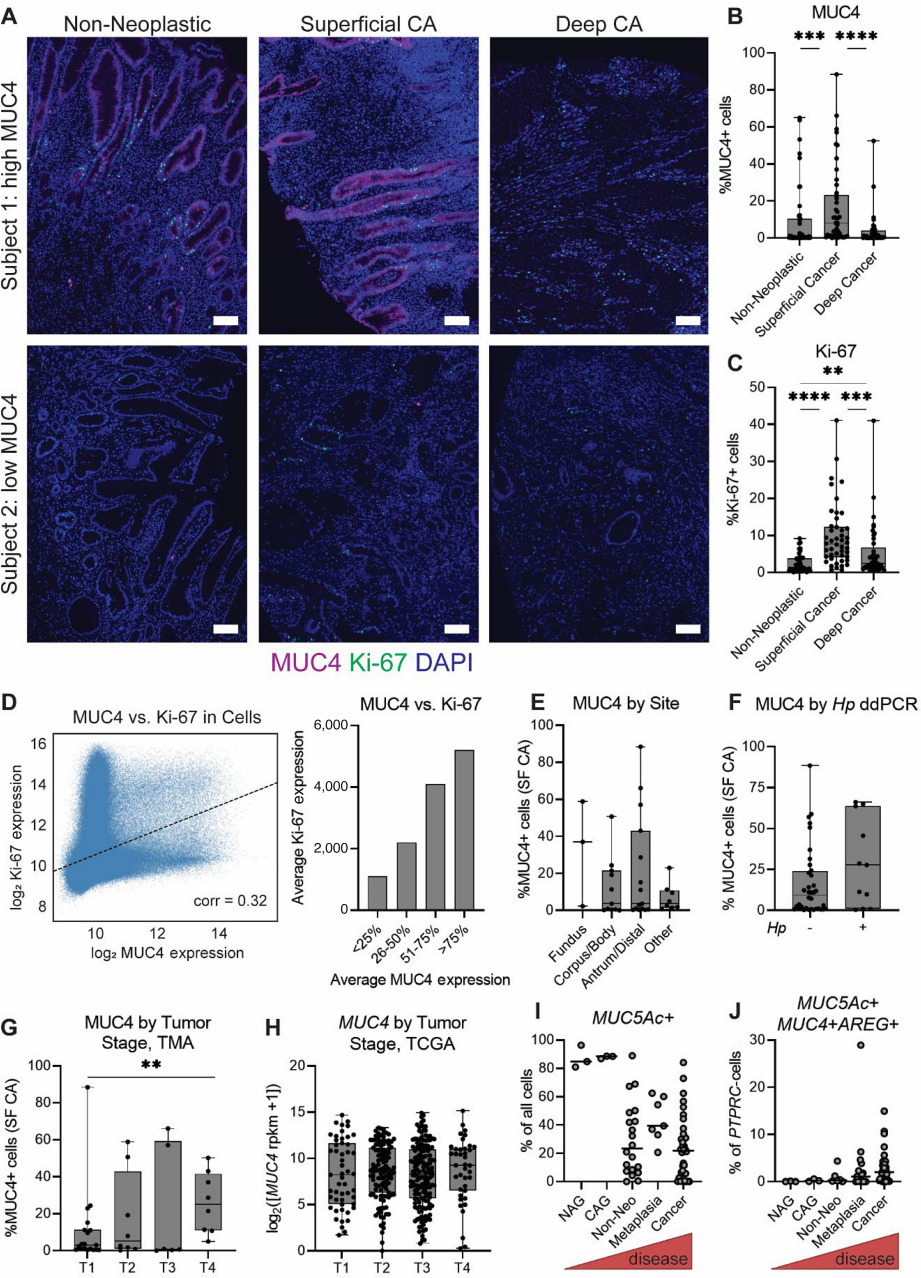
Metaplastic pit cells are detected in human subjects with preneoplasia and gastric cancer. **A–G,** The expression of MUC4 and the cell proliferation marker Ki-67 was probed in a TMA comprising samples from 47 gastric cancer (“CA”) patients. **A,** Marker expression is shown in tissue sections from two representative patients. MUC4 is shown in magenta, Ki-67 in green and nuclei (DAPI) in blue. Scale bars, 100 μm. **B–D,** QuPath was used to segment individual cells within each tissue core and determine their marker expression by pixel intensity. **B** and **C,** The percentage of positive cells was calculated for each marker and sample, and is shown as box-and-whisker plots overlaid with individual values for each sample. MUC4 is shown in B and Ki-67 in C. **, *P* < 0.01; ***, *P* < 0.001; ****, *P* < 0.0001, Mann–Whitney *U* test. **D,** MUC4 and Ki-67 were assessed within every cell in the TMA. The left image shows every cell on a representative slide. On the right, every cell in the slide was binned in quartiles according to its MUC4 expression level and the mean Ki-67 expression level of the cells in each quartile was calculated. Bars indicate the 95% confidence interval of the estimated true mean. **E–G,** The proportion of MUC4+ cells in the superficial samples (shown in B) was plotted against clinical parameters of the TMA subjects. **E,** MUC4 levels are reported according to the anatomic site of the cancer. “Other” denotes other gastric anatomical sites (e.g., gastroesophageal junction) and cases where the specific gastric site could not be determined from the available pathology notes (e.g., “diffuse”). **F,** Gastric *Hp* status was determined by ddPCR and subjects were categorized as *Hp*− or *Hp*+ to indicate whether their analyzed tissues still harbored active *Hp* infection. **G,** MUC4 levels are reported according tumor (“T”) stage. **, *P* < 0.01, Mann–Whitney *U* test for T1 versus T4. **H,***MUC4* transcript levels, reported as log_2_-transformed [*MUC4* reads per kilobase per million mapped reads (rpkm) +1], were assessed in TCGA and plotted according to tumor (“T”) stage. **I** and **J,** Three published gastric scRNA-seq datasets were mined for cell types of interest. Each dot represents one scRNA-seq sample, which are arranged in order of disease severity. **I,** Pit cells (*MUC5Ac*+) are shown as the proportion of all cells in each sample. NAG, non-atrophic gastritis; CAG, chronic atrophic gastritis. **J,** Metaplastic pit cells (*MUC5Ac*+*MUC4*+*AREG*+) are shown as the proportion of non-hematopoietic cells (*PTPRC*−) in each sample.

Gastric cancers often consist of mixed cell types including pit or pit-like cells (34, 35), but the significance of pit cells in disease development is not well understood. Thus, we used a tissue microarray (TMA) to assess MUC4 expression in samples from 47 recent gastric cancer cases seen at medical centers in the U.S. Pacific Northwest (Supplementary Table S6). Samples consisted of 2-mm tissue cores from regions of superficial cancer, deep cancer, and non-neoplastic epithelium adjacent to cancer, which were immunostained for MUC4 and the proliferation marker Ki-67 (Fig. 6A). We used QuPath to segment cells via nuclear staining and to quantify marker expression within each core based on fluorescence intensity (ref. 36; Supplementary Table S7). Marker expression differed according to tissue type (Fig. 6B and C). Both MUC4 and the proliferation marker Ki-67 had the highest expression in superficial cancer compared with the other tissues; as well, Ki-67 expression in deep cancer was higher than in non-neoplastic epithelium. To assess whether MUC4 and Ki-67 were coexpressed within individual cells (i.e., whether MUC4-expressing cells were proliferative), we probed their expression in each cell from all tissue cores, for a total of 2.69 million cells. MUC4 and Ki-67 were highly statistically significantly correlated, with a modest positive association (Fig. 6D; Supplementary Fig. S12). Thus, MUC4-expressing cells are likely to be a proliferative cell population in the stomach.

We next assessed whether MUC4 expression correlated with the demographic and clinical information available for the subjects in the TMA. For these analyses, we used the percent of MUC4+ cells in the superficial cancer samples, since these had the greatest MUC4 expression. MUC4 was not associated with sex or age (Supplementary Fig. S13). High MUC4 expression was observed in tumors from a variety of stomach sites, including fundus, corpus (body), and antrum (Fig. 6E), suggesting that metaplastic pit cells are not restricted to cancers from a specific gastric region. To determine whether MUC4 expression was correlated with active *Hp* infection, we used droplet digital PCR (ddPCR) to detect *Hp* in DNA extracted from the tissues used to generate the TMA. Eleven of 47 subjects had detectable *Hp* DNA, consistent with previous observations that at least half of patients with gastric cancer clear *Hp* infection prior to cancer diagnosis (37–39). The median level of MUC4 expression was higher in *Hp*+ subjects than *Hp*− subjects, though the difference was not statistically significant (Fig. 6F). Finally, in our cohort, MUC4 was associated with tumor stage, with stage 4 tumors having significantly higher MUC4 expression than stage 1 tumors (Fig. 6G). However, in a large dataset of gastric cancer samples from The Cancer Genome Atlas (TCGA; ref. 40), *MUC4* was not associated with tumor stage (Fig. 6H), which could be due to differences in demographics between TCGA cohort and our subjects, and/or different assays of gene expression (bulk vs. single cell). Thus, gastric MUC4 expression occurs in a variety of tumor sites, may be somewhat more common in samples with active *Hp* infection, and may be associated with tumor stage in some cohorts.

### Metaplastic Pit Cells Increase in Number as Gastric Disease Progresses

Finally, to explore how pit cells change over the course of human disease, we mined data from three recently published gastric scRNA-seq studies (Fig. 6I and J). One study had 9 subjects with 13 antral biopsies: three from regions of non-atrophic gastritis (NAG, mild gastric inflammation), three of chronic atrophic gastritis (CAG, loss of parietal and chief cells due to *Hp*-mediated inflammation), six of metaplasia and one of cancer (17). The other two studies comprised 20 samples from 7 subjects with cancer and one with metaplasia (18) and 48 samples from 31 subjects with cancer (19); these studies also included paired samples from non-neoplastic epithelium in the same subject. We observed that *MUC5Ac*+ cells, presumably pit cells, comprised a large proportion of cells sequenced in NAG and CAG samples, that is, mild disease. As disease severity increased, the proportion of *MUC5Ac+* (pit) cells detected from each sample decreased (Fig. 6I). Cells expressing *MUC5Ac*, *MUC4*, and *AREG*, that is, metaplastic pit cells, made up an increasing proportion of non-hematopoietic cells (*PTPRC*−) as disease progressed (Fig. 6J). Among the samples from later stages of disease, these cells comprised 0.03%–4.4% (median 0.29%) of non-hematopoietic cells in samples of metaplasia, 0%–28.9% (median 1.1%) of non-hematopoietic cells in non-neoplastic tissue adjacent to cancer, and 0%–14.9% (median 2.0%) of non-hemopoietic cells in cancer samples. Finally, we confirmed in our TMA that in human subjects with gastric cancer, pit cells (expressing *MUC5Ac*) could express *MUC4* (Supplementary Fig. S14). Thus, metaplastic pit cells increase in abundance with increasing gastric disease severity.

## Discussion

Up to half of gastric tumors harbor active *Hp* infection (37–39), and eradication of *Hp* in combination with surgical tumor resection significantly reduces cancer recurrence, compared with surgical resection without *Hp* eradication (14). In addition, eradication of *Hp* in subjects with metaplasia significantly reduces their risk of developing gastric cancer (12, 13). Cumulatively, these observations suggest that active *Hp* infection during the later stages of disease promotes cancer development. Here we used a mouse model of genetically-driven gastric intestinal metaplasia to test how the additional perturbation of chronic *Hp* infection impacts disease. We found that the combination of *Hp* infection and metaplasia driven by induction of a constitutively active *Kras* allele (*Hp*+KRAS+ mice) elicited a striking expansion of metaplastic cells most similar to surface mucus-producing pit cells. Metaplastic pit cells expressed the intestinal mucin *Muc4* and the EGFR ligand amphiregulin and were dependent on gastric inflammation. Thus, metaplasia in *Hp*+KRAS+ mice is not simply accelerated beyond what is seen in *Hp*−KRAS+ mice, but instead develops along an altered trajectory.

Gastric tumors often harbor pit or pit-like cells (34, 35) but the role of pit cells in preneoplasia and tumorigenesis is not well understood. A meta-analysis found that during gastric cancer, decreased expression of the pit cell mucin MUC5Ac was associated with deeper tumor penetration and worse overall survival (41). Here we found that *MUC5Ac*+ cells decreased in overall abundance as disease progressed in three scRNA-seq studies of human samples. However, as normal pit cells decreased in abundance, the proportion of pit cells expressing the intestinal mucin *MUC4* increased. Previous studies observed MUC4 in human gastric tumors but did not examine cell type–specific expression (42, 43). As a transmembrane mucin, MUC4’s extracellular domain can interact with the extracellular environment (which could include interactions with pathogens such as *Hp*), while its cytoplasmic domain can promote oncogenic signaling through multiple pathways, including HER2 (ErbB2) interactions and activation of Notch3 signaling (44). Our gene set enrichment analysis implicated ErbB signaling, which could involve both HER2 and ErbB1 (EGFR), and we found that the expression of *Muc4* was highly correlated with the expression of the EGFR ligand amphiregulin. Because amphiregulin can function in autocrine, paracrine, and juxtacrine fashion (45), induction of *Areg* in metaplastic pit cells could potentially lead to widespread changes in the stomach through paracrine signaling. As there are now several therapies available to target EGFR signaling during cancer, more work is needed to clarify the role(s) of amphiregulin in our model and in gastric cancer.

We found that MUC4 was significantly positively associated with the cell proliferation marker Ki-67 in individual cells from human subjects with gastric cancer. However, *Hp*+KRAS+ mice did not develop gastric tumors within a 3- to 4-month experimental time frame (15). Thus, metaplastic pit cells may serve as a noncancerous precursor cell type that is poised to become cancerous in certain contexts. Pit cell hyperplasia has been seen in other KRAS G12D models in the absence of *Hp* infection (46–48). Interestingly, *Tff1-Kras* mice had robust MUC4 expression in the gastric epithelium and increased expression of *Areg* compared with healthy control mice. Thus, multiple gastric perturbations can lead to MUC4 and amphiregulin expression, suggesting that this may be a conserved response to gastric injury.

*Hp* infection is known to promote pit cell (foveolar) hyperplasia as well as changes in pit cell gene expression and phenotypes (49, 50). Intriguingly, laser-capture microdissection of pit cells from *Hp*-infected Balb/c mice showed a signature of IFNγ response and actin rearrangement (49), two key responses that were upregulated in metaplastic pit cells compared with classical pit cells by pathway analysis of differential gene expression. The cytokine IFNγ has been shown to induce MUC4 expression in cultured cell lines, both individually (51) and synergistically with TNFα (52), and was correlated with metaplastic pit cell expansion in our mice. Our finding that dexamethasone treatment prevented metaplastic pit cell expansion supports a role for *Hp*-mediated inflammation in driving this cell type. In this study we focused on macrophages and T cells, not only because these immune cell populations were significantly correlated with metaplastic pit cell development but also because these cells have been associated with gastric disease in other animal models (3, 11, 33, 53). However, dexamethasone is broadly immunosuppressive and we cannot rule out the contribution of other immune cell types. Future work is needed to identify which immune cell population(s) and effector cytokines drive metaplastic pit cell expansion. Intriguingly, long-term use of non-steroidal anti-inflammatory drugs is associated with reduced risk of noncardia gastric cancer (54).

Our study has several limitations. (i) It may be that the specific changes in disease marker expression observed in *Hp*+KRAS+ mice are due to effects on *Mist1*-expressing isthmal progenitors (55) in addition to, or instead of, effects on chief cells. Although we cannot rule out that possibility, we note that a study that used the highly chief cell-specific reporter GIF (gastric intrinsic factor) confirmed that chief cells are the primary cell of origin of SPEM (4). Future studies will test whether metaplastic pit cells are found in other genetically engineered mouse models of gastric intestinal metaplasia and/or cancer. (ii) Gastric cancer is quite heterogeneous and RAS pathway activity is only found in approximately 40% of tumors (40). However, we and others previously showed that induction of constitutively active KRAS in *Mist1*-expressing cells is sufficient to drive metaplasia and dysplasia, thus establishing that constitutively active KRAS is a tool to broadly model aspects of human disease. Future studies will address whether metaplastic pit cells are only found in subjects with RAS pathway activity or may be more widespread. (iii) We observed a trend toward increased MUC4 expression in human subjects with active *Hp* infection (by ddPCR) in their analyzed tissues. However, only 11 of 47 subjects had evidence of *Hp* by ddPCR, limiting the statistical power of these analyses. We note that about 80% of gastric cancers are attributed to *Hp* infection, so it is likely that the majority of our subjects had an *Hp* infection that was either cleared prior to cancer diagnosis or was below our limit of detection. In a previous study with 63% *Hp* positivity, *Hp* was significantly positively associated with the expression of the intestinal mucins MUC2 and MUC4 in gastric tumors (56).

Our mouse model provides a means to dissect the cell lineages that arise during infection-associated preneoplastic progression. In the current study, we discovered that *Hp* infection during gastric intestinal metaplasia leads to the expansion of *Muc4*-expressing metaplastic pit cells. More work is needed to determine whether *Muc4* expression is the driver of pit cell metaplasia, or whether *Muc4* may be a passenger in another gene expression program that drives disease development; indeed, metaplastic pit cells express other metaplasia- and cancer-related genes. In published single sequencing data from human subjects, *MUC4* expression was greatest in pit cells from subjects with metaplasia and cancer. Most patients with metaplasia do not progress to cancer, but MUC4 expression was strongly linked with Ki-67 expression in gastric cancer tissues in our human cohort. Future studies will test whether the presence of metaplastic pit cells during gastric intestinal metaplasia may serve as a risk factor for progression from metaplasia to cancer.

## Supplementary Material

Supplementary Methods Expanded Materials and Methods.

Table S1 Description of the mouse samples used for single-cell RNA-sequencing.

Table S2 Genes driving the clustering of cells in UMAP #1; genes with power ≥ 0.4 are given.

Table S3 Genes driving the clustering of cells in UMAP #2. Up to the top 70 genes with the highest fold change and Padjusted < 0.05 are given for each cluster.

Table S4 The 10 most differentially expressed genes within each cluster of UMAP #2.

Table S5 Pathway analysis of metaplastic pit cells (pit_2, pit_6 and pit_8) versus classical pit cells (pit_1, pit_3, pit_4, pit_5 and pit_7) from UMAP #2.

Table S6 Characteristics of human subjects used in this study.

Table S7 Expression of the mucin MUC4 and the cell proliferation marker Ki-67 in each specimen in the gastric cancer tissue microarray (TMA).

Table S8 List of antibodies, lectins and probes used in this study.

Figure S1 Summary of cell clusters detected in different mouse treatment groups at six and 12 weeks.

Figure S2 Cell cluster frequencies change according to Hp infection status and induction of constitutively active KRAS.

Figure S3 Genes driving the subclustering of the major gastric epithelial cell types.

Figure S4 The epithelial subclusters pit_2, pit_6, pit_8 and neck_2 are expanded in Hp+KRAS+ mice.

Figure S5 The dysplasia marker gene Trop2 is rarely detected in the central epithelial megacluster, whereas Muc4 and Areg are primarily detected in the central epithelial megacluster.

Figure S6 Spatial profiling reveals enrichment of Muc4 and Areg expression from the luminal surface in the Hp+KRAS+ gastric epithelium.

Figure S7 Metaplastic pit cells expand within six weeks and are partially dependent on the Hp cag type IV secretion system.

Figure S8 IFN-γ but not IL-4 is associated with metaplastic pit cell expansion.

Figure S9 Gating strategy for detection of myeloid cell populations by flow cytometry.

Figure S10 Gating strategy for detection of T cell populations by flow cytometry.

Figure S11 Gastric inflammation is greatest in Hp+KRAS+ mice.

Figure S12 MUC4 and Ki-67 expression are significantly positively associated in samples from 47 subjects with gastric cancer.

Figure S13 MUC4 expression was not associated with sex or age in our cohort of 47 gastric cancer cases seen in the United States Pacific Northwest.

Figure S14 Figure S14. Metaplastic pit cells can be seen in gastric cancer samples.
