# Supplementary material for: Single-cell Profiling Uncovers a Muc4-Expressing Metaplastic Gastric Cell Type Sustained by Helicobacter pylori-driven Inflammation
Source: Cancer Res Commun. 2023 Sep 5;3(9):1756–69. doi: 10.1158/2767-9764.CRC-23-0142 (PMC10478791; doi:10.1158/2767-9764.CRC-23-0142)
Supplement: Supplementary Methods — Expanded Materials and Methods. [file crc-23-0142-s01.pdf]

Supplemental Methods

Data availability statement

Single-cell RNA-sequencing data was deposited in GEO under the project accession number GSE224840. The accession numbers for the individual samples are: GSM7034070, GSM7034071, GSM7034072, GSM7034073, GSM7034074, GSM7034075, GSM7034076 and GSM7034077. Other data generated in this study are available upon request from the corresponding author.

Mouse model of gastric preneoplasia

All mouse experiments were approved by the Fred Hutchinson Cancer Center Institutional Animal Care and Use Committee (protocol number 1531) and were performed in accordance with the recommendations in the National Institutes of Health Guide for the Care and Use of Laboratory Animals. *Mist1-CreERT2 Tg/+, LSL-Kras (G12D) Tg/+* ("*Mist1-Kras*") mice were previously described [1,2]. Male and female mice aged eight to 16 weeks old were randomized to treatment groups. On day one, mice were infected with *Hp* strain PMSS1 [3] or mock-infected with broth. On days two through four, mice were given 5 mg tamoxifen (Sigma) in corn oil (Sigma) by subcutaneous injection, or were given corn oil alone. Mice were humanely euthanized after two, six or 12 weeks and tissues were analyzed.

Bacterial infections

Helicobacter pylori strain PMSS1 or an isogenic Δ*cagE* mutant was cultured and mice were inoculated with 10^8 CFU as previously described [1]. "Triple therapy" antibiotic treatments were previously described [1]. For immunosuppression with oral corticosteroids, because the Fred Hutch small animal vivarium acidifies its water to pH ~3.5, all mice were given autoclaved, non-acidified water starting at two weeks after infection and constitutively active KRAS induction. Dexamethasone (Sigma) was added to the drinking water at 1 mg/liter. Control animals received non-acidified water only. Water was protected from light and changed weekly and mice were euthanized at six weeks.

scRNA-seq of the gastric corpus

Stomachs were aseptically harvested. One third was removed and fixed in 10% neutral-buffered formalin. The remainder of the tissue was trimmed to remove the forestomach and antrum and transferred to ice-cold PBS. Tissues were washed by agitating in three serial passages of ice-cold PBS, then single-cell suspensions were generated using digestion at 4°C with protease from *Bacillus licheniformis* (Sigma P5380). An established protocol was used (see [4] section 3.2 – Adult Mouse Lung) with the following modifications: instead of weighing 25 mg of tissue, stomach tissue was minced and then divided in half, and each half was used in a protease digestion reaction; samples were rocked gently on a rocker in between trituration steps; and a cell strainer was not used as straining led to epithelial cell loss. After single cell suspensions were generated, some samples were subjected to cryopreservation. Briefly, cells were stored in Recovery Cell Culture Freezing Medium (Gibco), cooled slowly overnight in a CoolCell Container (BioCision) placed at -80°C, then stored long-term in liquid nitrogen. Cells were rapidly thawed in a 37°C water bath. At the 6 week time point, cells were thawed from one *Hp*-KRAS+ mouse and one *Hp*+KRAS+ mouse. At the 12 week time point, thawed cells were pooled from 2-3 mice per treatment group (described in **Table S1**). Dead cells were removed using magnetic bead separation (MACS Dead Cell Removal Kit with MS columns, Miltenyi Biotec). Live cells were pelleted, resuspended in phosphate-buffered saline with 0.04% non-

acetylated bovine serum albumin, and counted with a hemacytometer using Trypan Blue (0.4%, Gibco) to distinguish between live (clear) and dead (blue) cells.

In an attempt to increase the number of sequenced cells, two samples from the 12 week time point were not subjected to cryopreservation. Instead, the single cell suspensions were generated from one *Hp*-KRAS+ and one *Hp*+KRAS+ mouse using cold protease digestion as described above, then dead cells were immediately removed using magnetic bead separation as described above. The live cells were then counted and immediately used.

Whether samples were cryopreserved or not, after dead cell clean up, cells were used for gel beads-in-emulsion (GEM) generation and barcoding using the Chromium Next GEM Single Cell 3' Reagent Kits v3.1 and Nextera library preparation (both from 10x Genomics) according to the manufacturer's instructions. After standard library quality control metrics, samples were sequenced in a HiSeq rapid flow cell with an Illumina HiSeq 2500 sequencer (six week time point) and a NovaSeq SP 100 flow cell with an Illumina NovaSeq 6000 sequencer (12 week time point).

scRNA-seq analysis

Quality control and dimension reduction

Sequencing reads were aligned to the mouse genome using cellRanger (10x Genomics). Seurat was applied to filter the output feature count matrixes of these samples to include only cells expressing at least 250 genes and genes expressed in at least 10 cells (18,089 genes and 22,050 cells passed this filter from a starting population of 6.7 million GEMs), and to filter out cells with >25% mitochondrial content (16,434 cells remained after this filter). Filtered cells were integrated into a single dataset using the default Seurat parameters. The integrated reads were normalized, scaled and subjected to principal component analysis (PCA) dimension reduction. A SNN clustering method was applied on the processed dataset to find 25 clusters using the first 20 dimensions after PCA reduction and resolution of 0.5. Clusters were then visualized on a 2-dimensional UMAP plot. We applied Seurat's FindAllMarkers function to identify the representative marker genes for each cluster and assigned cell types accordingly. To explore the predominant gastric epithelial cell populations, cells from the central epithelial "megacluster" in the 12 week samples were separated and re-clustered into 14 clusters using the same Seurat processing workflow described above, with a resolution of 0.7. To generate forest plots and heat maps showing cell abundance, samples were categorized according to treatment and time (i.e., the two 12 week *Hp*-KRAS+ and the two 12 week *Hp*+KRAS+ samples were each condensed into one sample). For each group, the proportion and confidence interval of each cell type was estimated from the empirical Bayesian distribution based on the observations of cell type occurrence in R using the EBBR package. The corresponding forest plots and heat maps were generated using ggplot2. Forest plot error bars represent the confidence interval that a given cell would be identified as a given cell type based on the observed cell distributions in the dataset. Heatmaps showing gene expression in different cell types and conditions were generated in Python via the Pandas and Seaborn packages using the average value of normalized gene expression of all cells from a given cell type or condition. Contour plots for *Muc5ac* and *Muc4* expression were generated with the Seaborn package using scaled and normalized gene expression data from Seurat.

Gene Set Enrichment Analysis

We applied the FindAllMarkers function from Seurat (minimum 10% cells expressing that gene, minimum $\log_2$ fold change of 0.25 between the two groups) to achieve a list of differentially expressed genes between clusters pit_2, pit_6 and pit_8 (metaplastic pit cells) vs. pit_1, pit_3,

pit_4, pit_5 and pit_7, using cells from all mouse groups. Statistically significant genes (false discovery rate [FDR] <= 0.05, Wilcoxon rank sum test) were used as the input for downstream GSEA (Gene Set Enrichment) analysis (GSEA Pre-ranked, fold change value as ranking order) using Hallmark, KEGG and GO pathway datasets from MSigDB [5,6]. A 0.15 FDR value was used as the cutoff for statistically significantly enriched pathways.

Spatial gene expression (10x Visium)

Four mice were used: *Hp*-KRAS-, *Hp*+KRAS-, *Hp*-KRAS+ and *Hp*+KRAS+, each at the 12 week time point. Mouse stomachs were embedded in OCT and snap-frozen in isopentane/liquid nitrogen, then stored at -80°C. Using a cryotome, sections of 10 µm thickness were cut onto a Visium slide (10x, serial number V11F01-280) with two sections (from the same mouse) per capture area. Adjacent sections were cut onto glass slides and stained with Diff-Quick (Differential Quik III Stain Kit, Polysciences) to assess tissue morphology. On the day of the experiment, the slide was fixed and stained according to the manufacturer's instructions. After washing, the slide was mounted in 85% glycerol (Fisher) with 2 U/µl Protector RNase inhibitor (Millipore Sigma) and imaged on a Leica Microsystems DMi8 scanning microscope. Tissue sections were permeabilized for six minutes and RNA was extracted and libraries prepared according to the manufacturer's instructions. The libraries were pooled with another Visium sample and sequenced on a NovaSeq SP 100 flow cell with an Illumina NovaSeq 6000 sequencer, then deconvoluted post-sequencing. The multicolor LIF and TIFF images were viewed in SpaceRanger (10x Genomics) and fiducial markings were used to align the Visium spots. Regions of folded or damaged tissue were discarded. The cleaned data was log-normalized and PCA-reduced based on highly variable genes with BayesSpace [7] using the default settings. The number of clusters for each sample was defined as the elbow point of the qPlot, using the first 15 dimensions of the PCA. Feature expression plots were generated using the BayesSpace-enhanced data via the enhanceFeature function from BayesSpace.

Analysis of published human scRNA-seq datasets

We collected three human scRNA datasets from subjects with gastric pre-cancer or early gastric cancer [8-10] to validate the existence of metaplastic pit cells in the human population. The feature count matrix from each subject was fed into the standardized Seurat processing pipeline. Sequencing data were filtered by Seurat default settings to remove cells with high mitochondrial mRNA amounts or low gene expression. Filtered data were normalized and scaled and the number of cells expressing a certain gene was determined by the subset function in R.

Flow Cytometry

Stomachs were aseptically harvested, the antrum and forestomach were discarded, and the remaining corpus tissue was kept on ice in RP-3: RP-0 (RPMI medium [Gibco] containing 1% penicillin/streptomycin/L-glutamine [Gibco] and 1% HEPES [Cytiva]), with the addition of 3% HyClone fetal bovine serum (Cytiva). Tissues were opened, rinsed with a basic buffer (10 mM HEPES, pH 8.2) and cut into 3-4 pieces, which were incubated at 400 rpm with stirring for 20 minutes at 37°C in 10 ml RP-3 with 5 mM EDTA (Sigma) and 0.15 g/L DTT (Sigma). Tissue pieces were vigorously shaken three times for 30 seconds each in 7 ml RP-0 with 0.5M EDTA and strained over a kitchen strainer to remove epithelial cells and intraepithelial lymphocytes. The tissue pieces were finely minced with scissors, then incubated at 400 rpm with stirring for 25 minutes at 37°C in 5-7 ml of a digestion buffer of RP-0 with 10 µg/ml DNase I (Roche) and 0.2 mg/ml Liberase TL (Roche). The enzymatic reaction was stopped with the addition of 10 ml cold RP-3 and tissues were filtered through a 70 µm strainer. The remaining tissue pieces were gently mashed with the rubber end of a 1 ml syringe. Cells were pelleted and resuspended in 5 ml 37.5% Percoll (Cytiva), then centrifuged for 20 mins at room temperature in an Eppendorf

benchtop centrifuge (5810 R) at 1800 rpm with Ascend and Descend set to 1. The supernatant was aspirated and the pellet, containing lamina propria immune cells, was resuspended in 270 µl RP-3, of which 100 µl was used to detect myeloid cells and 100 µl was used to detect lymphocytes as follows. Briefly, cells were incubated in 40 µl of 1:1000 Ghost Dye R780 fixable viability dye (Tonbo). This and all subsequent steps were performed in the dark at room temperature unless otherwise stated. After 20 minutes, 10 µl of 1:400 Fc block was added (anti-mouse CD16/32, 2.4G2, BD). After 10 minutes, 50 µl of the extracellular antibody stain was added (see **Table S8**). After 30 minutes, cells were pelleted. Cells stained with the myeloid panel were resuspended in 200 µl of 2% paraformaldehyde. Cells stained with the lymphocyte panel were resuspended in 200 µl of Fix/Perm (FoxP3/Transcription Factor Staining Buffer Set, eBioscience, diluted according to manufacturer's instructions) and incubated for 20 minutes at 4°C. Cells were pelleted, washed in 200 µl PermWash (eBioscience, diluted according to manufacturer's instructions) and incubated with 100 µl of intracellular antibody stain (see **Table S8**) in PermWash for 30 minutes. Cells were pelleted and resuspended in 200 µl FACS buffer (PBS with 2% fetal bovine serum and 1 mM EDTA). Finally, 50 µl of the 270 µl cell preparation was stained with 1:1000 Ghost Dye (R780) and fixed with 2% paraformaldehyde containing 20,000 counting beads (Accucheck Counting Beads, Invitrogen), and used to determine the number of viable cells per sample. Cells were stored at 4°C for 1-2 days and then run on a BD FACSymphony A5 High-Parameter Cell Analyzer. Beads (UltraComp eBeads, Invitrogen) stained with each individual antibody were used for compensation, with the exception of the viability dye control, where compensation was performed with leftover cells incubated with the viability dye alone. Data were analyzed in FlowJo_v10.8.1.

Cytokine Analysis

Mice were euthanized as described above and one third of the stomach (from limiting ridge to antrum) was homogenized and serially diluted to culture *Hp*. The homogenate was pelleted at 20,000 rcf for 5 minutes and supernatants were stored at -20°C. To measure IL-4 and IFN-γ, supernatants were thawed and tested without dilution using a BD Cytometric Bead Array (CBA) Mouse Th1/Th2/Th17 Cytokine Kit according to the manufacturer's instructions. Samples were run on a BD FACSymphony A5 High-Parameter Cell Analyzer and data were analyzed in FlowJo_v10.8.1.

Gastric cancer TMA

This study was approved by the Fred Hutchinson Cancer Center Institutional Review Board (IR8657). All procedures were conducted in accordance with recognized ethical guidelines of the Declaration of Helsinki, Belmont Report and U.S. Common Rule. A database search was performed to identify subjects with gastric cancer who had tissues stored at the University of Washington Northwest BioSpecimen tissue repository, Seattle, WA, or stored at the Legacy Research Institute Tumor Bank, Portland, OR (the latter accessed through the Fred Hutch Specimen Acquisition Network) since 2010. Subjects were excluded if they had neoadjuvant therapy or insufficient stored materials. A pathologist (LK) reviewed the medical records to determine which tissue blocks to request. Available blocks were pulled and sectioned as follows: one 4 µm cut that was stained with hematoxylin and eosin (H&E), followed by three 10 µm unstained cuts, then one 4 µm H&E-stained cut. DNA was extracted from 1-2 unstained cuts using the AllPrep DNA/RNA FFPE kit (Qiagen). DNA concentrations were determined by spectrophotometry with a NanoDrop One. Twenty µl of undiluted DNA was used to perform droplet digital PCR to detect the *H. pylori* 16S rRNA gene as previously described [11,12]. Samples were tested in duplicate. Samples with 0 copies/µl DNA were considered negative. Samples with 1-2 copies/µl DNA were re-extracted and re-tested. The second H&E-stained slide was then annotated by a pathologist (CY) to note regions of superficial cancer, deep cancer and

non-neoplastic tissue adjacent to cancer. The annotated H&E slides were used to select coring regions within the corresponding paraffin blocks and generate a tissue microarray (TMA) using TMA Grand Master (3DHISTECH) according to the manufacturer's instructions. TMA blocks were then sectioned (4 μm) onto ProbeOn Plus slides (Fisher) and stained for immunofluorescence microscopy as described below.

In situ *hybridization and immunofluorescence microscopy*

Immunofluorescence microscopy to detect gastric MUC4 and Ki-67 was performed on fixed tissue as previously described [1]. Multiplex immune cell immunohistochemistry studies to detect macrophages and T cells were performed as previously described [1]. *In situ* hybridization was performed on fixed tissue sections using the RNAscope system (ACD-biotechne) in accordance with the manufacturer's instructions, using a Leica Bond RX autostainer. The RNAscope 2.5 LS Reagent Kit – BROWN was used for *Muc4* alone and the RNAscope LS Multiplex Fluorescent Assay Kit was used to assess *Muc4* and *Areg* together. Briefly, paraffin-embedded tissue sections were baked at 65°C for 60 minutes, then deparaffinized and hydrated on the Leica Bond RX autostainer. Heat-Induced Epitope Retrieval was performed with reagents from the indicated RNAscope reagent kits according to the manufacturer's instructions. Briefly, tissues were incubated in Tris/EDTA, pH 9.0, for 15 minutes at 95°C, followed by a protein digestion in protease III for 15 minutes at room temperature. ISH probes and amplification reagents were applied according to the manufacturer's instructions. After staining, slides were manually dehydrated through graded alcohols, cleared in xylene, and mounted in Epredia Cytoseal XYL (Fisher). A list of antibodies and RNA probes is given in **Table S8**.

Image Analysis

<u>Analysis of mouse tissues</u>

For multiplex immune cell immunohistochemistry studies, CD4, CD8α, and F4/80 were quantified as previously described using HALO software (Indica Labs) [1]. *Muc4* ISH was scored in a blinded fashion using a semi-quantitative scale with the following criteria: 0 = no staining, 1 = 1-25% of corpus glands are positive, 2 = 26-50% of corpus glands are positive, 3 = 51-75% of corpus glands are positive, 4 = >75% of corpus glands are positive. For samples at the six week time point, instead of an overall score, the median score of 4-5 fields of view is reported due to greater heterogeneity in *Muc4* expression within individual mice.

<u>Analysis of human tissues</u>

Immunostained TMA slides were scanned on a ScanScope FL slide scanner (Aperio Technologies) using the ScanScope Console.lnk software v120.0.0.33. Slide scans were converted to TIFF files for downstream analysis using Aperio Image Scope software v12.3.1.5011. The relationship between MUC4 and Ki-67 in each TMA tissue core was quantified using a single cell resolution measurement providing fractional values of single or double positive cells from the total number of cells in each sample. TIFFs were imported into the open-source software QuPath 0.3.2 [13], and single cells were segmented from the DAPI (nuclear) signal using a built-in segmentation algorithm. Cell boundaries were approximated by tessellation of the nuclear masks, and the mean of either nuclear pixel intensity (for Ki-67) or whole cell pixel intensity (for MUC4) was extracted. The percentage of single- or double-positive cells per TMA core was then assessed. Because fluorescence intensity varied from slide to slide, analysis of marker abundance was conducted per slide. For each slide, the three tissue cores with the lowest average marker value were identified and visually inspected to confirm that they had low to no marker expression. These cores were used to set a baseline for negative/background staining. The average marker value and standard deviation was calculated

for the three cores, and a threshold of five deviations above this baseline level was used as a cutoff for positive cells among all tissue cores on the given slide.

Supplemental References

1 O'Brien, V. P. *et al.* Sustained *Helicobacter pylori* infection accelerates gastric dysplasia in a mouse model. *Life Sci Alliance* **4**, doi:10.26508/lsa.202000967 (2021).

2 Choi, E., Hendley, A. M., Bailey, J. M., Leach, S. D. & Goldenring, J. R. Expression of Activated Ras in Gastric Chief Cells of Mice Leads to the Full Spectrum of Metaplastic Lineage Transitions. *Gastroenterology* **150**, 918-930 e913, doi:10.1053/j.gastro.2015.11.049 (2016).

3 Arnold, I. C. *et al.* Tolerance rather than immunity protects from *Helicobacter pylori*-induced gastric preneoplasia. *Gastroenterology* **140**, 199-209, doi:10.1053/j.gastro.2010.06.047 (2011).

4 Potter, A. S. & Steven Potter, S. Dissociation of Tissues for Single-Cell Analysis. *Methods Mol Biol* **1926**, 55-62, doi:10.1007/978-1-4939-9021-4_5 (2019).

5 Subramanian, A. *et al.* Gene set enrichment analysis: a knowledge-based approach for interpreting genome-wide expression profiles. *Proc Natl Acad Sci U S A* **102**, 15545-15550, doi:10.1073/pnas.0506580102 (2005).

6 Liberzon, A. *et al.* Molecular signatures database (MSigDB) 3.0. *Bioinformatics* **27**, 1739-1740, doi:10.1093/bioinformatics/btr260 (2011).

7 Zhao, E. *et al.* Spatial transcriptomics at subspot resolution with BayesSpace. *Nat Biotechnol* **39**, 1375-1384, doi:10.1038/s41587-021-00935-2 (2021).

8 Zhang, P. *et al.* Dissecting the Single-Cell Transcriptome Network Underlying Gastric Premalignant Lesions and Early Gastric Cancer. *Cell Rep* **27**, 1934-1947 e1935, doi:10.1016/j.celrep.2019.04.052 (2019).

9 Sathe, A. *et al.* Single-Cell Genomic Characterization Reveals the Cellular Reprogramming of the Gastric Tumor Microenvironment. *Clin Cancer Res* **26**, 2640-2653, doi:10.1158/1078-0432.CCR-19-3231 (2020).

10 Kumar, V. *et al.* Single-Cell Atlas of Lineage States, Tumor Microenvironment, and Subtype-Specific Expression Programs in Gastric Cancer. *Cancer Discov* **12**, 670-691, doi:10.1158/2159-8290.CD-21-0683 (2022).

11 Talarico, S. *et al.* Quantitative Detection and Genotyping of *Helicobacter pylori* from Stool using Droplet Digital PCR Reveals Variation in Bacterial Loads that Correlates with cagA Virulence Gene Carriage. *Helicobacter* **21**, 325-333, doi:10.1111/hel.12289 (2016).

12 Talarico, S. *et al.* High prevalence of *Helicobacter pylori* clarithromycin resistance mutations among Seattle patients measured by droplet digital PCR. *Helicobacter* **23**, e12472, doi:10.1111/hel.12472 (2018).

13 Bankhead, P. *et al.* QuPath: Open source software for digital pathology image analysis. *Sci Rep* **7**, 16878, doi:10.1038/s41598-017-17204-5 (2017).
