# Supplementary material for: Single-cell Profiling Uncovers a Muc4-Expressing Metaplastic Gastric Cell Type Sustained by Helicobacter pylori-driven Inflammation
Source: Cancer Res Commun. 2023 Sep 5;3(9):1756–69. doi: 10.1158/2767-9764.CRC-23-0142 (PMC10478791; doi:10.1158/2767-9764.CRC-23-0142)
Supplement: Table S8 — List of antibodies, lectins and probes used in this study. [file crc-23-0142-s09.pdf]

Table S8. List of antibodies and probes used in this study.

Immunohistochemistry of human tissues:

Marker	Species	Dilution	Source	Purpose
Ki-67	Rabbit	1:100	D2H10; Cell Signaling Technology	Proliferating cell marker
MUC4	Mouse	1:1000	8G7, Surinder K. Batra (UNMC)	Metaplasia marker

Secondary antibodies used at 1:500 dilution:

Host	Reactive Against	Flour	Source
Donkey	Rabbit	AF488	Invitrogen
Donkey	Mouse	AF647	Invitrogen

RNAscope® 2.5 LS probes from ACD-biotechne used for *in situ* hybridization:

Target	Catalog Number
Mouse *Muc4*	534018
Mouse *Areg*	430508-C3
Human *MUC4*	312888
Human *MUC5Ac*	312898-C2

Antibodies and Opal dyes used for mouse immune cell immunohistochemistry:

Position	Antibody	Clone & Host	Manufacturer & Catalog Number	Dilution (Conc.)	Secondary	Opal Dye
1	CD3	SP7 Rabbit	Thermo RM-9107-S	1:400	Powervision Rabbit-HRP	Opal 520
2	F4/80	D2S9R Rabbit	Cell Signaling 20076S	1:4000 (0.1 µg/ml)	Powervision Rabbit-HRP	Opal 540
3	CD4	4SM95 Rat	eBioscience 14-9766-32	1:250 (2 µg/mL)	ImmPress Rat-HRP	Opal 570
4	CD8α	4SM15 Rat	eBioscience 14-0808-82	1:1000 (0.5 µg/ml)	ImmPress Rat-HRP	Opal 780

Antibodies used for flow cytometry:

Target	Fluor	Clone	Catalog Number	Manufacturer	Dilution
B220	BV510	RA3-6B2	103248	BioLegend	1:200
CD103	A488	2E7	121420	BioLegend	1:300
CD103	BUV737	M290	741739	BD	1:200
CD11b	BV605	M1/70	101257	BioLegend	1:400
CD11c	PeCy7	N418	25-0114-82	eBioscience	1:300
CD4	PC-eF710	GK1.5	46-0041-82	eBioscience	1:200
CD44	AF700	IM7	103026	BioLegend	1:200
CD45	BUV395	30-F11	564279	BD	1:250
CD64	PE	X54-5/7.1	139304	BioLegend	1:64
CD8α	BUV395	53-6.7	563786	BD	1:200
CD90.2	BV510	30-H12	105335	BioLegend	1:200
F4/80	BV650	T45-2342	743282	BD	1:200
FoxP3	BV421	FJK-16s	48-5773-82	eBioscience	1:150
Ly6C	BV711	HK1.4	128037	BioLegend	1:400
Ly6G	BUV563	1A8	612921	BD	1:400
MHC-II	PC-eF710	M5/114.15.2	46-5321-82	eBioscience	1:500
NK1.1	BV510	PK136	108738	BioLegend	1:400
TCRβ	BV650	H57-597	109251	BioLegend	1:200
TCRγδ	FITC	GL3	118128	BioLegend	1:200
