# Supplementary material for: Single-cell Profiling Uncovers a Muc4-Expressing Metaplastic Gastric Cell Type Sustained by Helicobacter pylori-driven Inflammation
Source: Cancer Res Commun. 2023 Sep 5;3(9):1756–69. doi: 10.1158/2767-9764.CRC-23-0142 (PMC10478791; doi:10.1158/2767-9764.CRC-23-0142)
Supplement: Figure S1 — Summary of cell clusters detected in different mouse treatment groups at six and 12 weeks. [file crc-23-0142-s10.pdf]

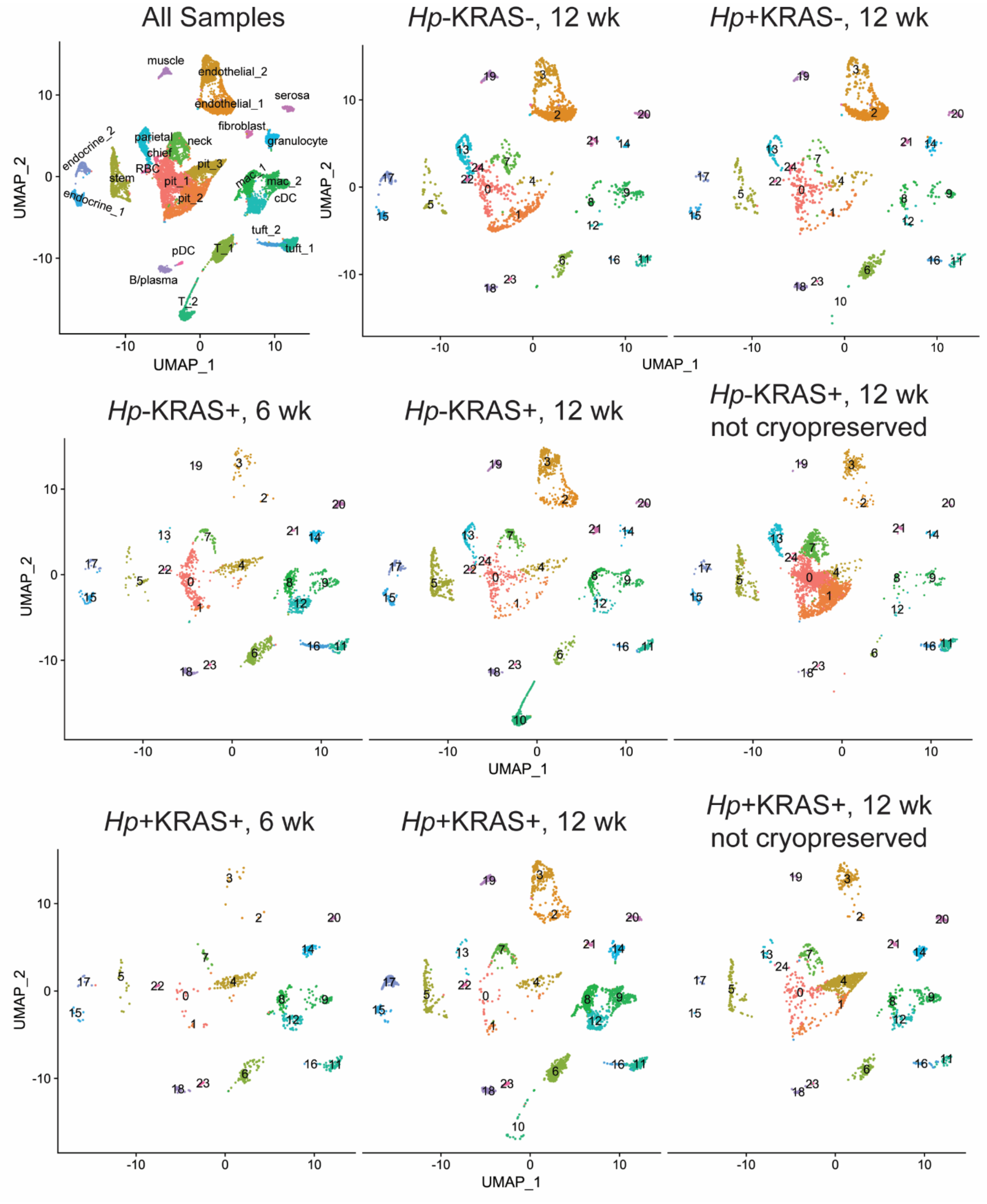

Figure S1. Summary of cell clusters detected in different mouse treatment groups at six and 12 weeks. Gastric single-cell RNA-sequencing was performed on eight samples obtained six or 12 weeks after *Hp* infection and/or constitutively active KRAS induction, and the UMAP from each sample is shown. Gastric single cell suspensions were prepared from mouse stomachs via digestion with a cold-active protease. Six of the samples were cryopreserved, then

thawed, pooled as indicated in Table S1, subjected to dead cell removal and captured in Gel Beads in Emulsion (GEMs) for sequencing. Two samples, indicated as “not cryopreserved,” were captured in GEMs immediately following single cell suspension generation and dead cell removal. The two libraries from the six week timepoint were barcoded and sequenced in one run and the six libraries from the 12 week timepoint were barcoded and sequenced in a second run, as described in Table S1. After standard quality control metrics, the number of cells per sample ranged from 723 (*Hp*+KRAS+, 6 wk) to 3574 (*Hp*-KRAS+, 12 wk, not cryopreserved). The UMAP #1 from Figure 1B is reproduced in the top left, and the other UMAPs correspond to the indicated treatment groups. Clusters were annotated as follows: 0, pit cell_1; 1, pit cell_2; 2, endothelial cell_1; 3, endothelial cell_2; 4, pit cell_3; 5, stem cell; 6, T cell_1; 7, mucous neck cell; 8, macrophage_1; 9, macrophage_2; 10, T cell_2; 11, tuft cell_1; 12, conventional dendritic cell; 13, parietal cell; 14, granulocyte; 15, enteroendocrine cell_1; 16, tuft cell_2; 17, enteroendocrine cell_2; 18, B or plasma cell; 19, muscle cell; 20, serosal cell; 21, fibroblast; 22, erythrocyte or reticulocyte; 23, plasmacytoid dendritic cell; 24, chief cell.
