# Supplementary material for: Single-cell Profiling Uncovers a Muc4-Expressing Metaplastic Gastric Cell Type Sustained by Helicobacter pylori-driven Inflammation
Source: Cancer Res Commun. 2023 Sep 5;3(9):1756–69. doi: 10.1158/2767-9764.CRC-23-0142 (PMC10478791; doi:10.1158/2767-9764.CRC-23-0142)
Supplement: Figure S2 — Cell cluster frequencies change according to Hp infection status and induction of constitutively active KRAS. [file crc-23-0142-s11.pdf]

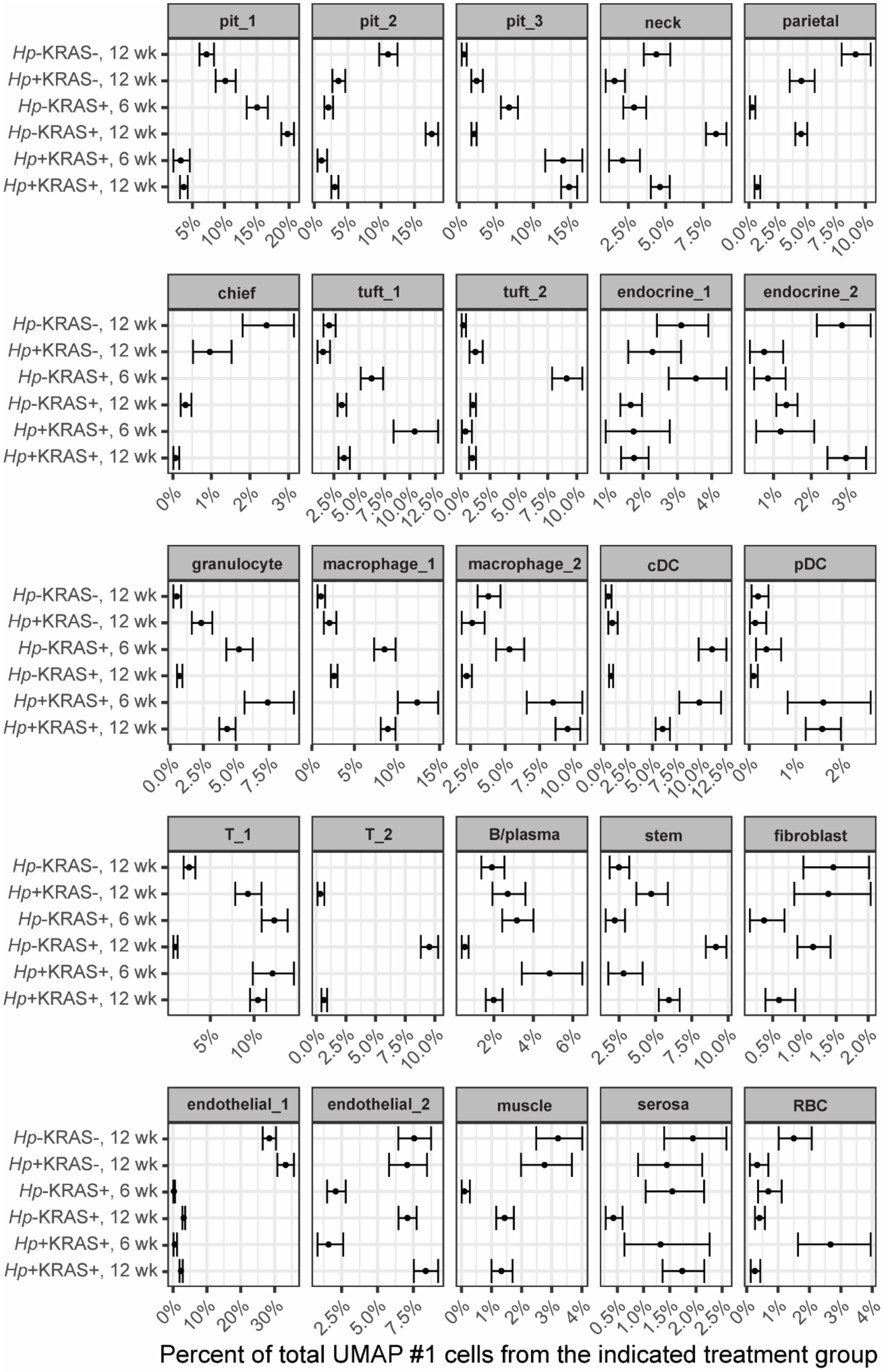

pit_1
pit_2
pit_3
neck
parietal
chief
tuft_1
tuft_2
endocrine_1
endocrine_2
granulocyte
macrophage_1
macrophage_2
cDC
pDC
T_1
T_2
B/plasma
stem
fibroblast
endothelial_1
endothelial_2
muscle
serosa
RBC
Hp-KRAS-, 12 wk
Hp+KRAS-, 12 wk
Hp-KRAS+, 6 wk
Hp-KRAS+, 12 wk
Hp+KRAS+, 6 wk
Hp+KRAS+, 12 wk
Percent of total UMAP #1 cells from the indicated treatment group

Figure S2. Cell cluster frequencies change according to *Hp* infection status and induction of constitutively active KRAS. The proportion of cells assigned to each annotated cluster in UMAP #1 is given. Each datapoint shows the total number of estimated cells of that subcluster for the given treatment group, reported as the percentage of all cells from that treatment group in UMAP #1. Samples were categorized according to treatment and time and the proportion and confidence level of each cell type was estimated from the empirical Bayesian distribution based on the observations of cell type occurrence using the EBBR package in R. Error bars represent the confidence interval that a given percentage of cells would be identified as the given type based on the distribution we observed. Endocrine, enteroendocrine cell; cDC, conventional dendritic cell; pDC, plasmacytoid dendritic cell, RBC, erythrocyte/reticulocyte.
