# Supplementary material for: Single-cell Profiling Uncovers a Muc4-Expressing Metaplastic Gastric Cell Type Sustained by Helicobacter pylori-driven Inflammation
Source: Cancer Res Commun. 2023 Sep 5;3(9):1756–69. doi: 10.1158/2767-9764.CRC-23-0142 (PMC10478791; doi:10.1158/2767-9764.CRC-23-0142)
Supplement: Figure S3 — Genes driving the subclustering of the major gastric epithelial cell types. [file crc-23-0142-s12.pdf]

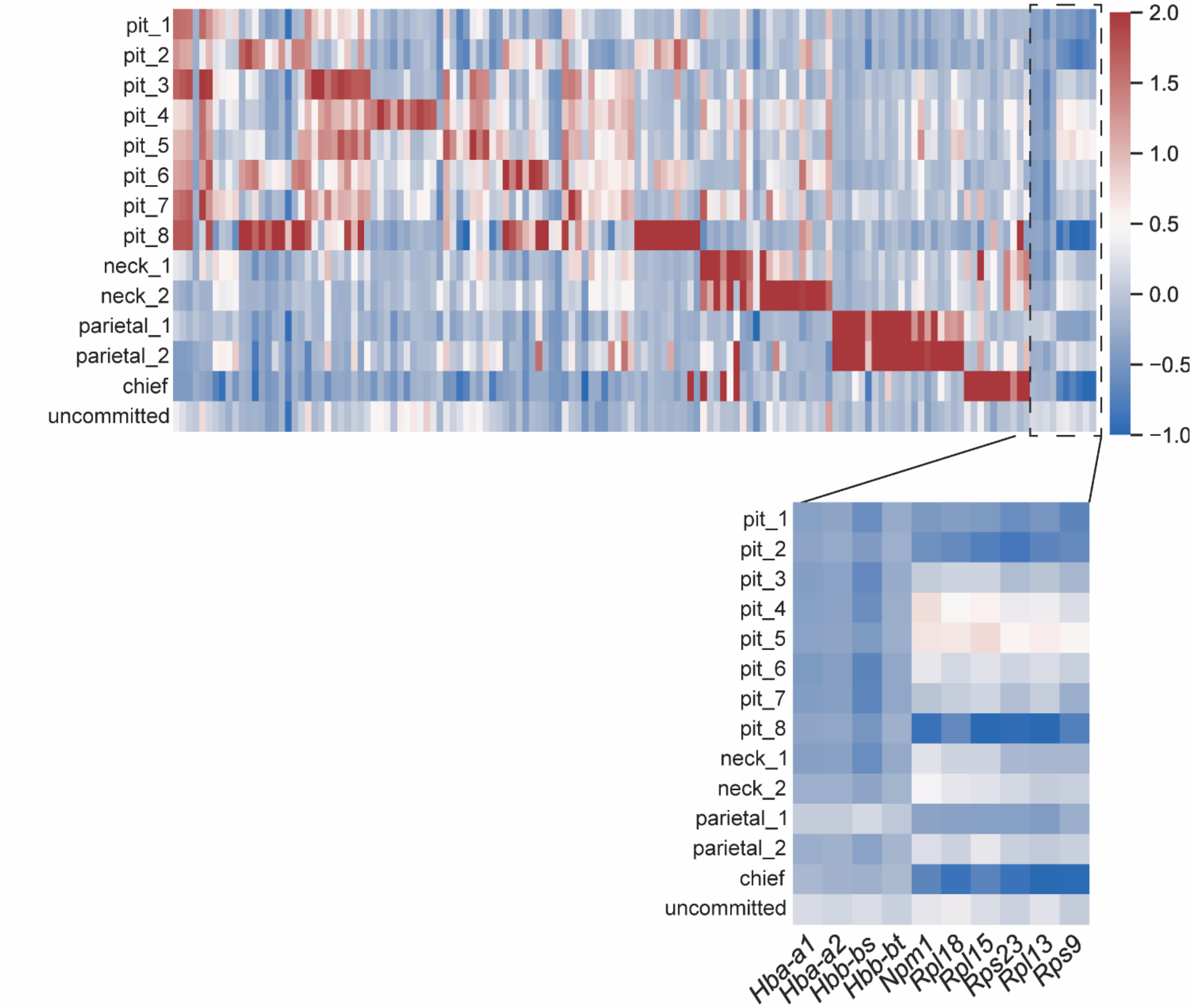

Figure S3. Genes driving the subclustering of the major gastric epithelial cell types. The heatmap shows the normalized expression of the top 10 genes driving the clustering of the indicated cell types from UMAP #2. The genes that segregate the “uncommitted” cluster (dashed line) are given. All genes in the heatmap are given in **Table S4**.
