# Supplementary material for: Single-cell Profiling Uncovers a Muc4-Expressing Metaplastic Gastric Cell Type Sustained by Helicobacter pylori-driven Inflammation
Source: Cancer Res Commun. 2023 Sep 5;3(9):1756–69. doi: 10.1158/2767-9764.CRC-23-0142 (PMC10478791; doi:10.1158/2767-9764.CRC-23-0142)
Supplement: Figure S4 — The epithelial subclusters pit_2, pit_6, pit_8 and neck_2 are expanded in Hp+KRAS+ mice. [file crc-23-0142-s13.pdf]

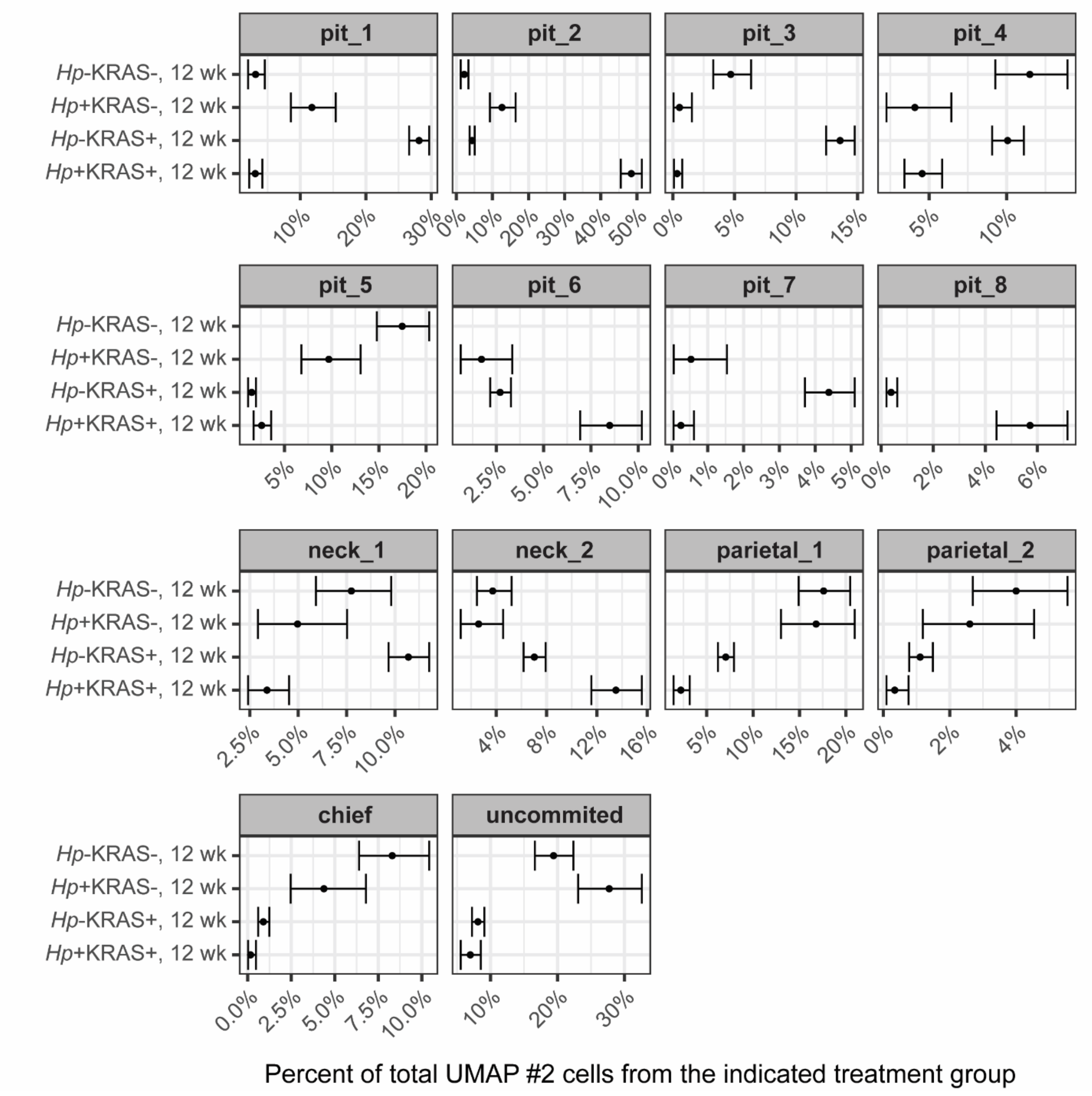

Figure S4. The epithelial subclusters pit_2, pit_6, pit_8 and neck_2 are expanded in *Hp*+KRAS+ mice. The proportion of cells assigned to each annotated cluster in UMAP #2 is given. Each datapoint shows the total number of estimated cells of that subcluster for the given treatment group, reported as the percentage of all cells from that treatment group in UMAP #2. Samples were categorized according to treatment and the proportion and confidence level of each cell type was estimated from the empirical Bayesian distribution based on the observations of cell type occurrence in R using the EBBR package. Error bars represent the confidence interval that a given percentage of cells would be identified as the given type based on the distribution we observed.
