# Supplementary material for: Single-cell Profiling Uncovers a Muc4-Expressing Metaplastic Gastric Cell Type Sustained by Helicobacter pylori-driven Inflammation
Source: Cancer Res Commun. 2023 Sep 5;3(9):1756–69. doi: 10.1158/2767-9764.CRC-23-0142 (PMC10478791; doi:10.1158/2767-9764.CRC-23-0142)
Supplement: Figure S5 — The dysplasia marker gene Trop2 is rarely detected in the central epithelial megacluster, whereas Muc4 and Areg are primarily detected in the central epithelial megacluster. [file crc-23-0142-s14.pdf]

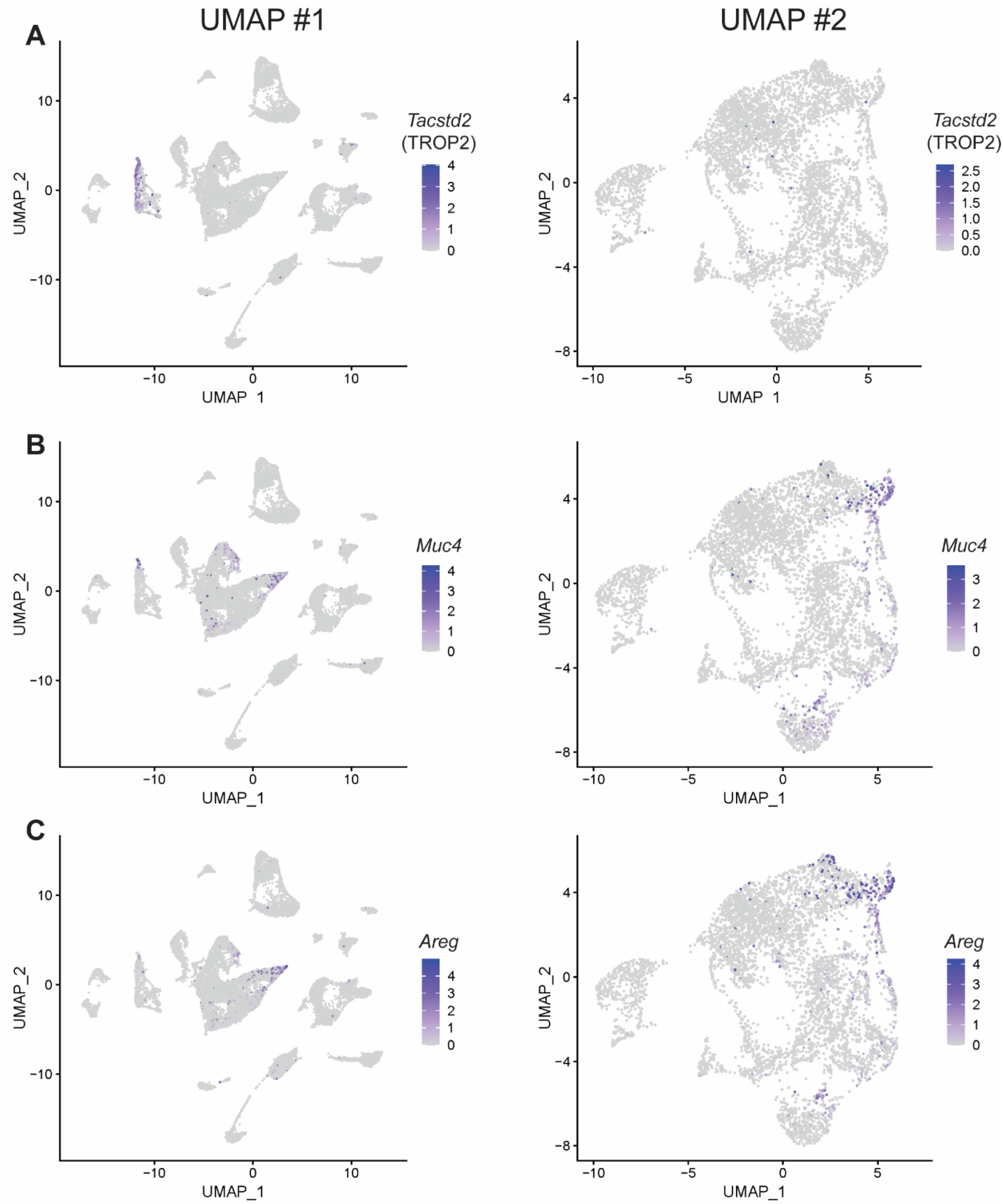

Figure S5. The dysplasia marker gene *Trop2* is rarely detected in the central epithelial megacluster, whereas *Muc4* and *Areg* are primarily detected in the central epithelial megacluster. Cells expressing the indicated genes are highlighted in purple on UMAP #1 (left) and UMAP #2 (right). The color scale indicates the magnitude of gene expression within a cell, expressed as ln([count per 10,000 reads]+1).
