# Supplementary material for: Single-cell Profiling Uncovers a Muc4-Expressing Metaplastic Gastric Cell Type Sustained by Helicobacter pylori-driven Inflammation
Source: Cancer Res Commun. 2023 Sep 5;3(9):1756–69. doi: 10.1158/2767-9764.CRC-23-0142 (PMC10478791; doi:10.1158/2767-9764.CRC-23-0142)
Supplement: Figure S6 — Spatial profiling reveals enrichment of Muc4 and Areg expression from the luminal surface in the Hp+KRAS+ gastric epithelium. [file crc-23-0142-s15.pdf]

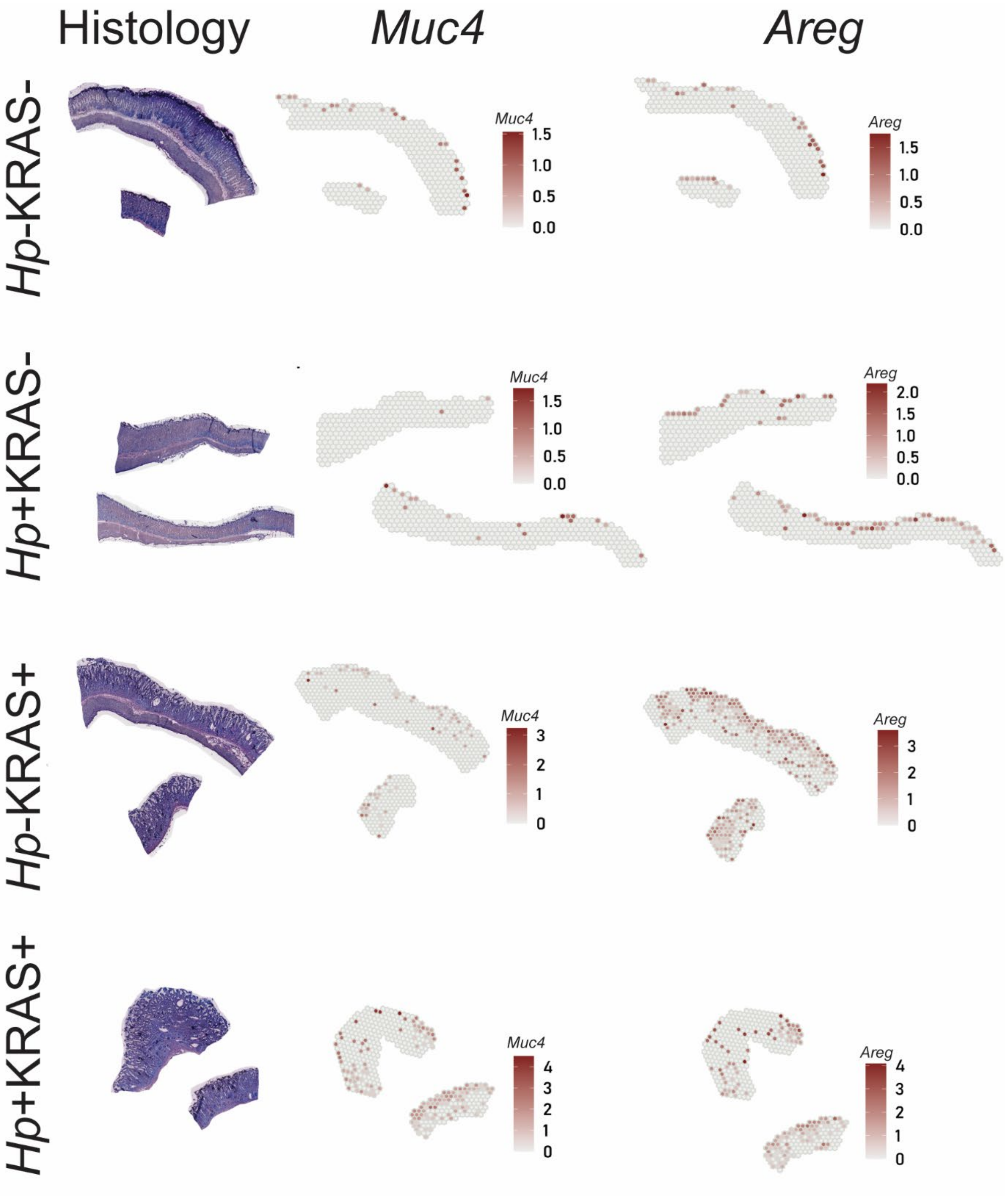

Figure S6. Spatial profiling reveals enrichment of *Muc4* and *Areg* expression from the luminal surface in the *Hp*+KRAS+ gastric epithelium. A spatial gene expression experiment conducted with the 10x Visium platform demonstrated greater metaplastic pit cell abundance in *Hp*+KRAS+ mice than in the other treatment groups. As described in the Supplemental Methods, at the 12 week time point, mice were humanely euthanized and tissues were cryo-preserved in OCT medium by plunge freezing in an isopentane/liquid nitrogen bath. Tissue sections were cut onto a barcoded Visium slide. After imaging, tissues were permeabilized to release RNA onto the barcoded spots. Libraries were prepared and sequenced and reads were

mapped back to the barcoded spots using SpaceRanger (10x). Damaged and folded regions of tissue were omitted from the analysis. Spots that were positive for *Muc4* and *Areg* are indicated. The proportion of *Muc4*+, *Areg*+, and *Muc4*+*Areg*+ spots was:

**Mouse**	**% *Muc4*+**	**% *Areg*+**	**% dual+**
Hp-KRAS-	6.3	9.1	3.3
Hp+KRAS-	3.3	12.1	1.4
Hp-KRAS+	10.6	38.4	7.5
Hp+KRAS+	31.9	29.3	17.8
