# Supplementary material for: Single-cell Profiling Uncovers a Muc4-Expressing Metaplastic Gastric Cell Type Sustained by Helicobacter pylori-driven Inflammation
Source: Cancer Res Commun. 2023 Sep 5;3(9):1756–69. doi: 10.1158/2767-9764.CRC-23-0142 (PMC10478791; doi:10.1158/2767-9764.CRC-23-0142)
Supplement: Figure S7 — Metaplastic pit cells expand within six weeks and are partially dependent on the Hp cag type IV secretion system. [file crc-23-0142-s16.pdf]

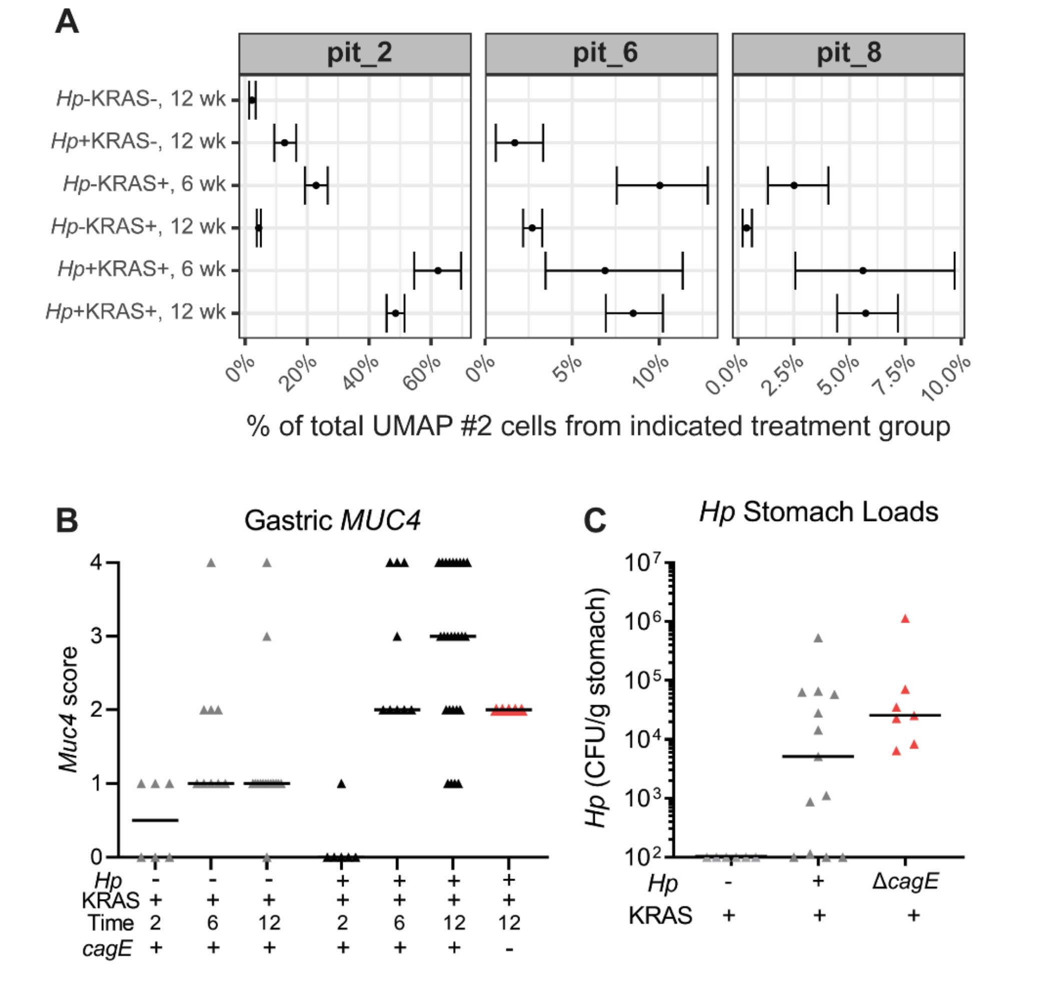

Figure S7. Metaplastic pit cells expand within six weeks and are partially dependent on the *Hp cag* type IV secretion system. A) The proportion of cells assigned to the indicated pit cell subclusters is shown for both the six and 12 week time points. Each datapoint shows the total number of estimated cells of that subcluster for the given treatment group, reported as the percentage of all cells from that treatment group in UMAP #2. Error bars represent the confidence interval that a given percentage of cells would be identified as the given cluster type based on their observed distribution. **B-C)** Mice were mock-infected or infected with wild-type *Hp* or an isogenic mutant (Δ*cagE*) that cannot assemble the *cag* type IV secretion system. All mice were given tamoxifen to induce constitutively active KRAS and mice were euthanized after two, six or 12 weeks. N=2-5 independent experiments were performed with n=3-8 mice per group. Data represent actual values from each individual mouse and bars indicate the median values. **B)** Tissues were scored for *Muc4* expression in a blinded fashion. **C)** Stomach titers of *Hp* are shown for the 12 week time point. CFU, colony-forming units. Zeroes are plotted at the limit of detection (100 CFU).
