# Supplementary material for: Single-cell Profiling Uncovers a Muc4-Expressing Metaplastic Gastric Cell Type Sustained by Helicobacter pylori-driven Inflammation
Source: Cancer Res Commun. 2023 Sep 5;3(9):1756–69. doi: 10.1158/2767-9764.CRC-23-0142 (PMC10478791; doi:10.1158/2767-9764.CRC-23-0142)
Supplement: Figure S8 — IFN-γ but not IL-4 is associated with metaplastic pit cell expansion. [file crc-23-0142-s17.pdf]

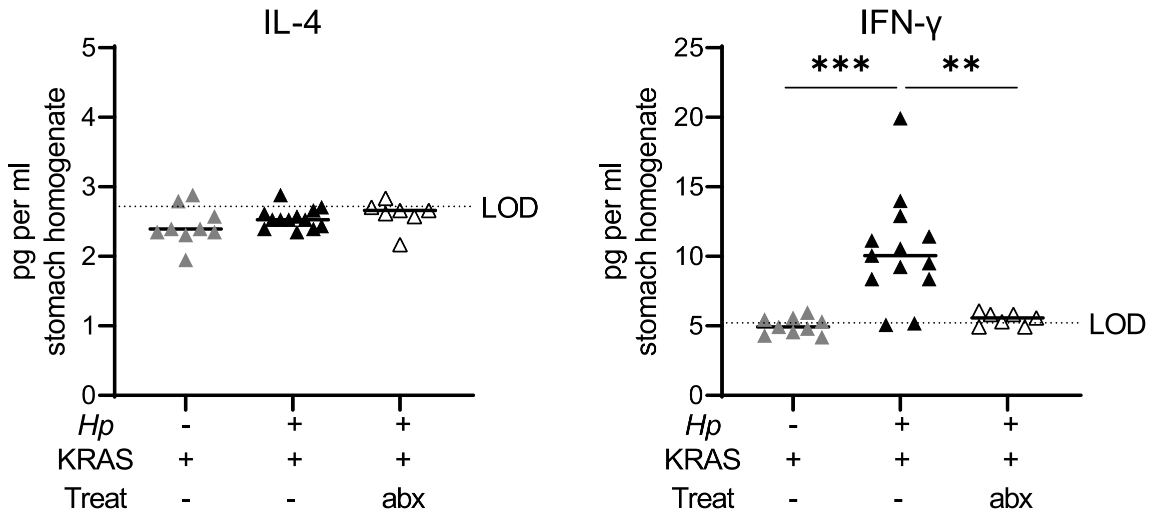

Figure S8. IFN-γ but not IL-4 is associated with metaplastic pit cell expansion. IL-4 and IFN-γ were measured in stomach homogenate supernatants from the indicated mouse treatment groups at a 12 week time point using a cytometric bead array. LOD, limit of detection; abx, antibiotics (*Hp*+KRAS+ mice that received antibiotic therapy to eradicate *Hp* from weeks six through eight; see Figure 4 in the main text).
