# Supplementary material for: Single-cell Profiling Uncovers a Muc4-Expressing Metaplastic Gastric Cell Type Sustained by Helicobacter pylori-driven Inflammation
Source: Cancer Res Commun. 2023 Sep 5;3(9):1756–69. doi: 10.1158/2767-9764.CRC-23-0142 (PMC10478791; doi:10.1158/2767-9764.CRC-23-0142)
Supplement: Figure S10 — Gating strategy for detection of T cell populations by flow cytometry. [file crc-23-0142-s19.pdf]

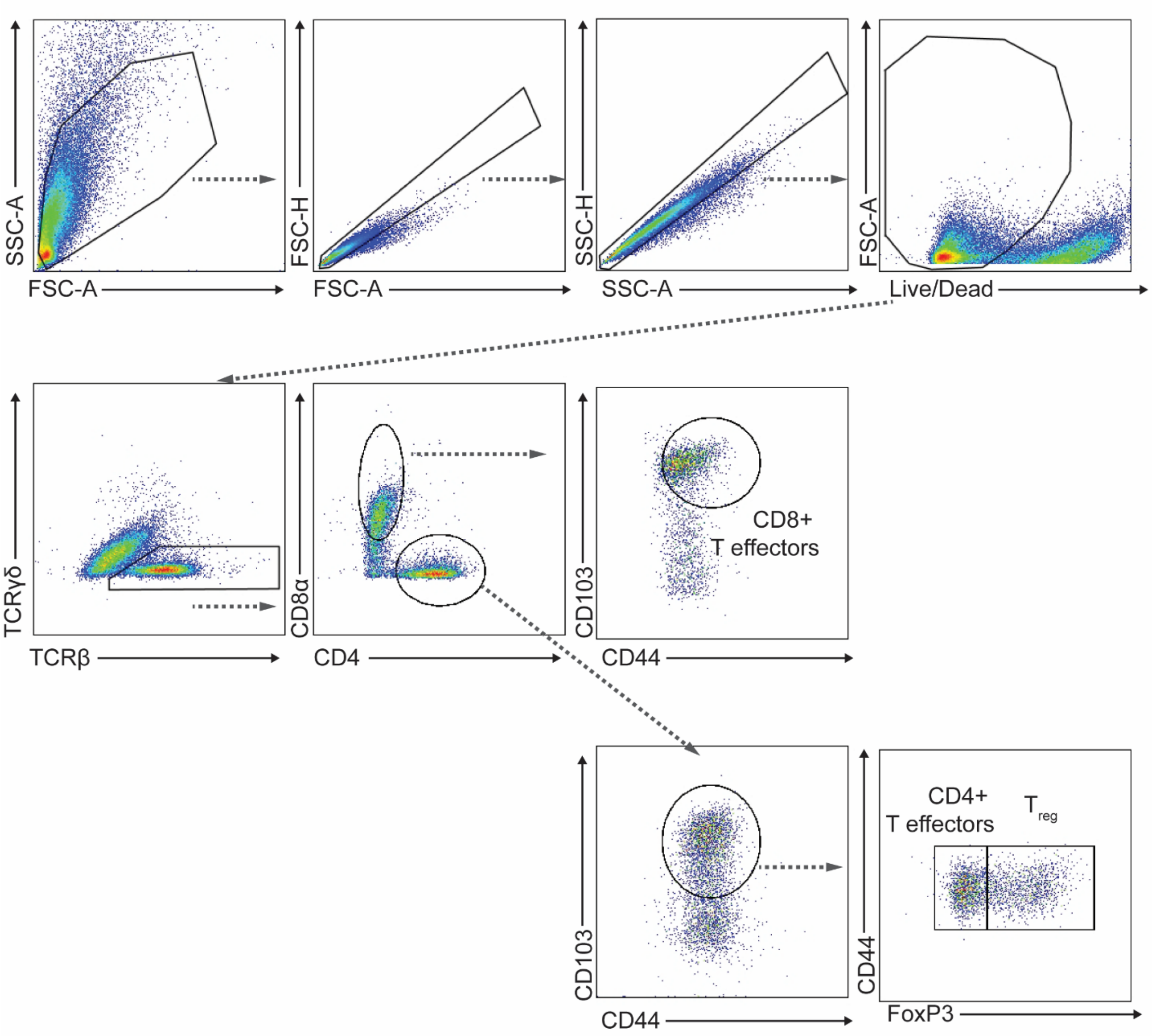

Figure S10. Gating strategy for detection of T cell populations by flow cytometry. Mouse gastric lamina propria cells were isolated and stained with the indicated markers. A representative *Hp*+KRAS+ mouse is shown.
