# Supplementary material for: Single-cell Profiling Uncovers a Muc4-Expressing Metaplastic Gastric Cell Type Sustained by Helicobacter pylori-driven Inflammation
Source: Cancer Res Commun. 2023 Sep 5;3(9):1756–69. doi: 10.1158/2767-9764.CRC-23-0142 (PMC10478791; doi:10.1158/2767-9764.CRC-23-0142)
Supplement: Figure S11 — Gastric inflammation is greatest in Hp+KRAS+ mice. [file crc-23-0142-s20.pdf]

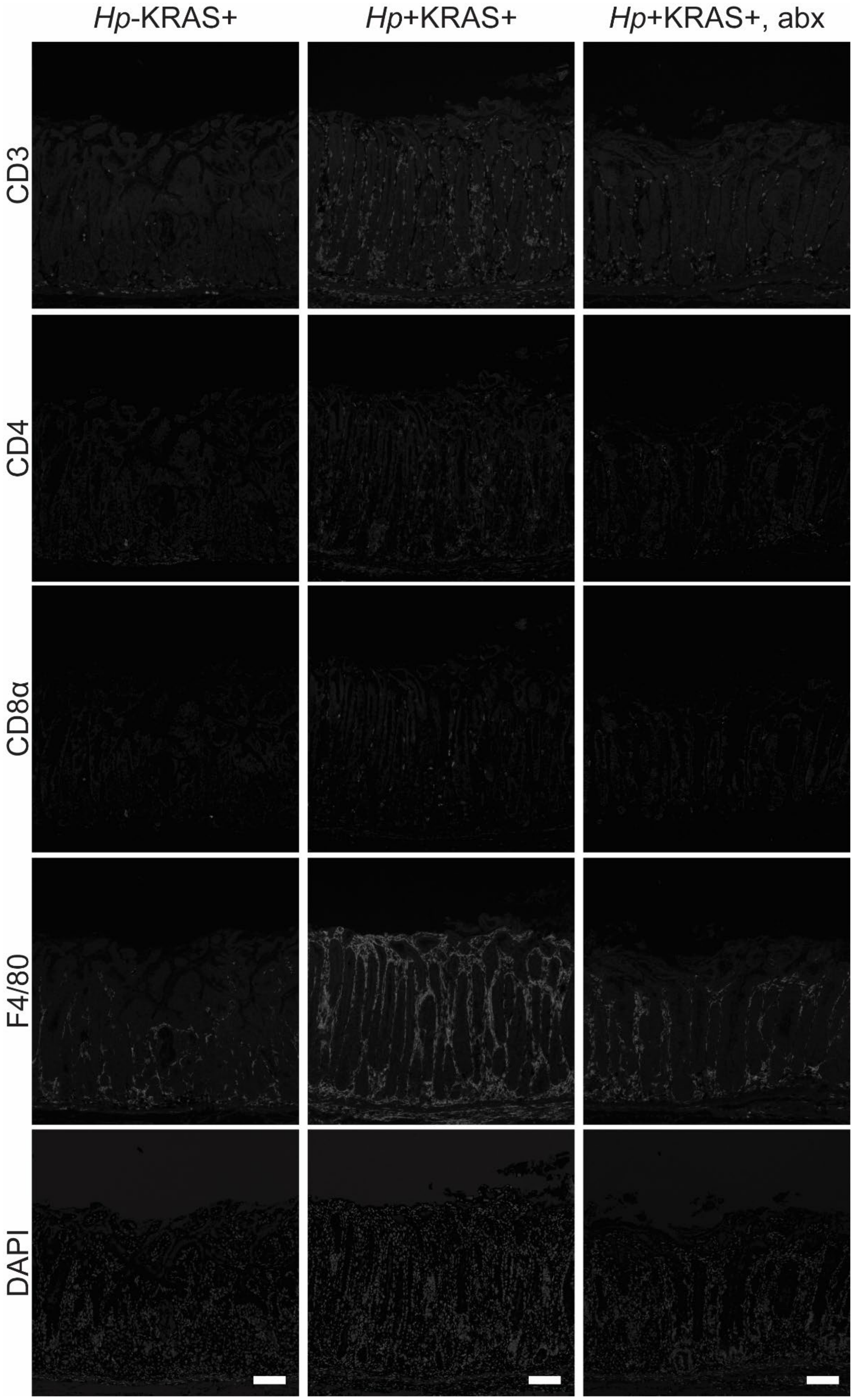

Hp-KRAS+
Hp+KRAS+
Hp+KRAS+, abx
CD3
CD4
CD8α
F4/80
DAPI

Figure S11. Gastric inflammation is greatest in *Hp*+KRAS+ mice. Gastric inflammation was assessed 12 weeks after KRAS induction in the following groups: *Hp*-KRAS+ mice; *Hp*+KRAS+ mice; and *Hp*+KRAS+ mice treated with antibiotics (‘abx’) from weeks six to eight. Shown are greyscale single-channel images from the composite representative images in Figure 4B in the main text. CD3 indicates T cells, CD4 indicates helper T cells, CD8α indicates cytotoxic T cells, F4/80 indicates macrophages and DAPI indicates nuclei. Scale bars, 100 μm.
