# Supplementary material for: Single-cell Profiling Uncovers a Muc4-Expressing Metaplastic Gastric Cell Type Sustained by Helicobacter pylori-driven Inflammation
Source: Cancer Res Commun. 2023 Sep 5;3(9):1756–69. doi: 10.1158/2767-9764.CRC-23-0142 (PMC10478791; doi:10.1158/2767-9764.CRC-23-0142)
Supplement: Figure S12 — MUC4 and Ki-67 expression are significantly positively associated in samples from 47 subjects with gastric cancer. [file crc-23-0142-s21.pdf]

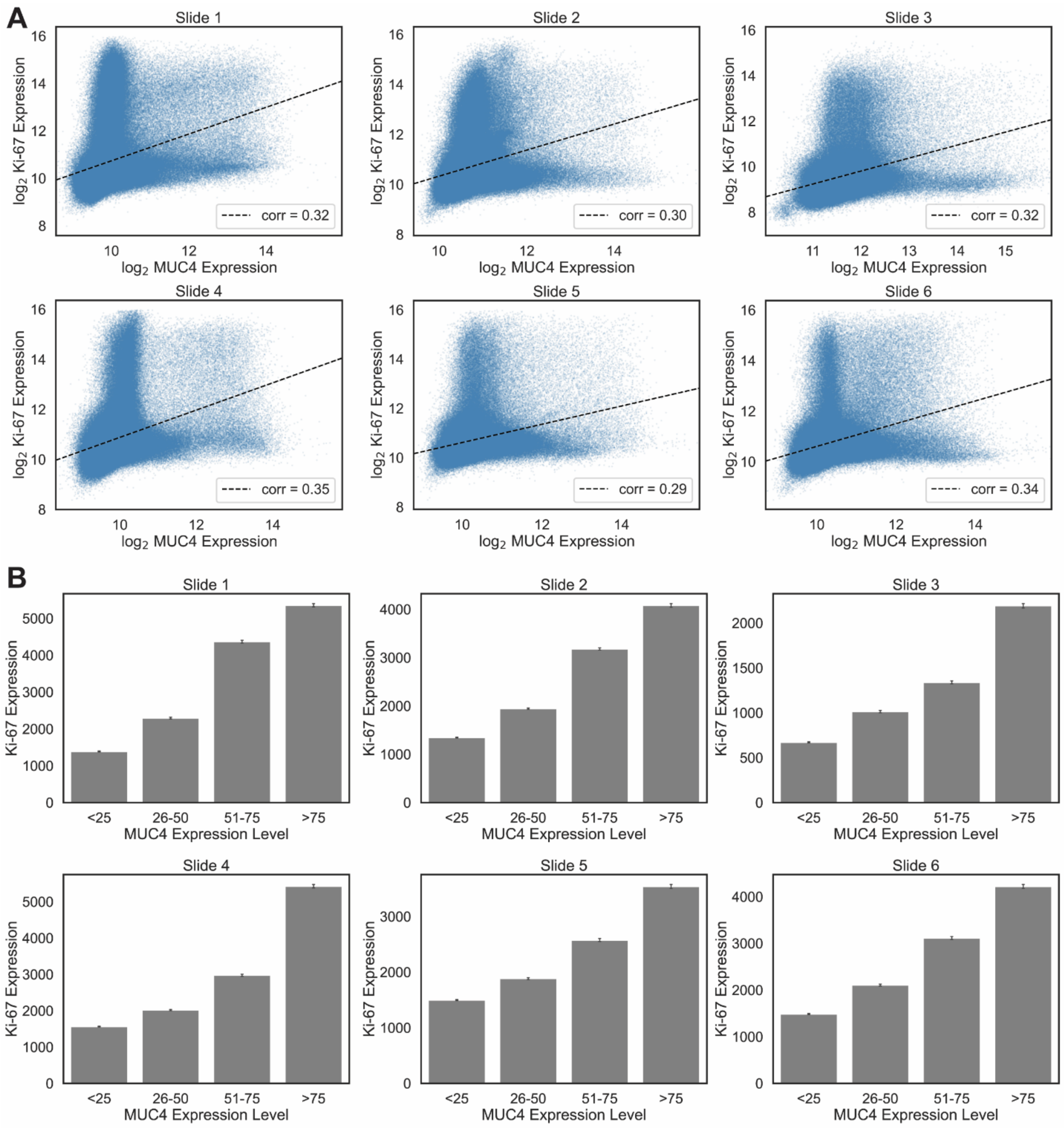

Figure S12. MUC4 and Ki-67 expression are significantly positively associated in samples from 47 subjects with gastric cancer. Samples were arranged in tumor microarrays (TMAs) comprising six paraffin blocks with 19-24 tissue cores per block. Each block was sectioned onto slides that were immunostained for MUC4 and Ki-67 expression and scanned for image analysis. Due to variability in staining intensity among the different slides, each slide was analyzed individually. QuPath was used to segment cells and detect marker expression within each cell of a given tissue core based on nuclear (Ki-67) and cellular (MUC4) signal intensity in the corresponding channels. **A)** The log_2-transformed signal intensity of MUC4 and Ki-67 is plotted for every cell in every tissue core on each individual slide. The following numbers of cells were detected: slide 1, 506,369; slide 2, 477,241; slide 3, 344,500; slide 4, 499,623; slide 5, 438,598; slide 6, 424,600 (total n=2,690,991 cells). Statistical significance was tested with a

Pearson's correlation and the correlation coefficient ("corr") is given; for each slide, $P < 0.00001$. **B)** For each slide, every cell in every tissue core was binned in quartiles according to its MUC4 expression level and the mean Ki-67 expression level of the cells in each quartile was calculated. Bars indicate the 95% confidence interval of the estimated true mean. Scatter and bar plots were made using the Seaborn package in Python. Slide 1 is reproduced in Figure 6 in the main text.
