# Supplementary material for: Single-cell Profiling Uncovers a Muc4-Expressing Metaplastic Gastric Cell Type Sustained by Helicobacter pylori-driven Inflammation
Source: Cancer Res Commun. 2023 Sep 5;3(9):1756–69. doi: 10.1158/2767-9764.CRC-23-0142 (PMC10478791; doi:10.1158/2767-9764.CRC-23-0142)
Supplement: Figure S13 — MUC4 expression was not associated with sex or age in our cohort of 47 gastric cancer cases seen in the United States Pacific Northwest. [file crc-23-0142-s22.pdf]

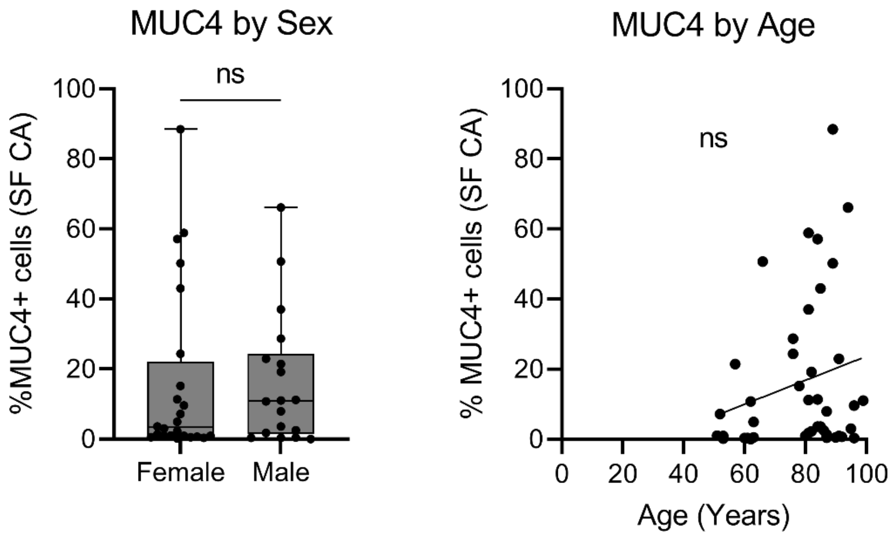

Figure S13. MUC4 expression was not associated with sex or age in our cohort of 47 gastric cancer cases seen in the United States Pacific Northwest. The expression of MUC4 was probed in a tissue microarray (TMA) comprising samples from 47 subjects with gastric cancer. QuPath was used to segment individual cells within each tissue core and determine their marker expression based on pixel intensity, and the proportion of MUC4+ cells in the superficial cancer ('SF CA') samples, shown in **Figure 6B** in the main text, was plotted against the subjects' sex and age at time of tumor collection, if known. Subjects missing the relevant demographic information were omitted from the graphs. Statistical significance was assessed by a Mann-Whitney U test for sex and by a Pearson correlation for age; n.s., not significant.
