# Supplementary material for: Single-cell Profiling Uncovers a Muc4-Expressing Metaplastic Gastric Cell Type Sustained by Helicobacter pylori-driven Inflammation
Source: Cancer Res Commun. 2023 Sep 5;3(9):1756–69. doi: 10.1158/2767-9764.CRC-23-0142 (PMC10478791; doi:10.1158/2767-9764.CRC-23-0142)
Supplement: Figure S14 — Metaplastic pit cells can be seen in gastric cancer samples. [file crc-23-0142-s23.pdf]

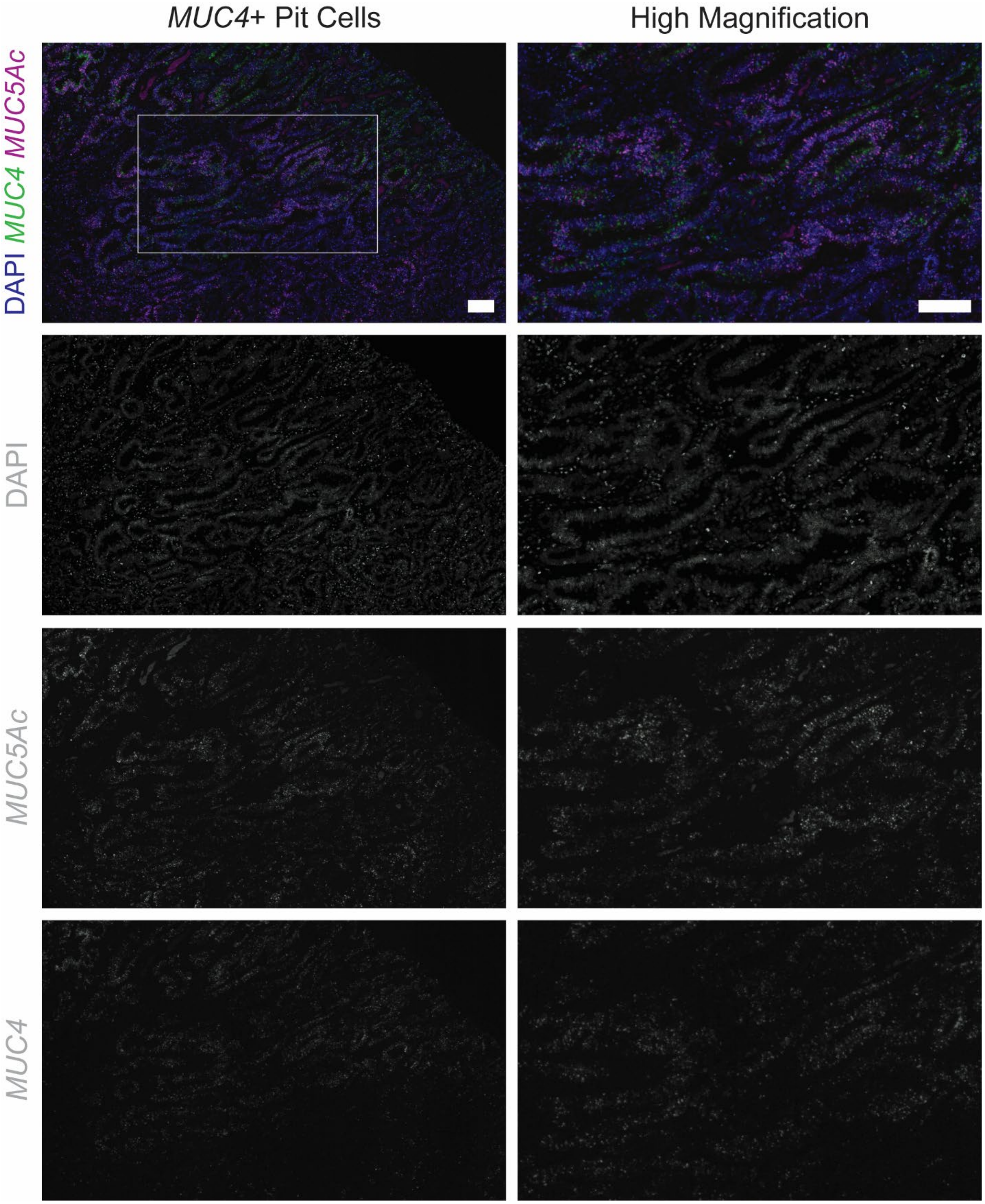

Figure S14. Metaplastic pit cells can be seen in gastric cancer samples. *In situ* hybridization was performed to detect the classical pit cell mucin *MUC5Ac* (magenta) and the intestinal mucin *MUC4* (green). Nuclei were stained with DAPI (blue). Shown is a representative image from a superficial cancer sample. The box indicates the region shown at higher magnification on the right. Scale bars, 100 μm.
